# Computational Investigation of Structural Dynamics of SARS-CoV-2 Methyltransferase-Stimulatory Factor Heterodimer nsp16/nsp10 Bound to the Cofactor SAM

**DOI:** 10.3389/fmolb.2020.590165

**Published:** 2020-11-24

**Authors:** Md Fulbabu Sk, Nisha Amarnath Jonniya, Rajarshi Roy, Sayan Poddar, Parimal Kar

**Affiliations:** Discipline of Biosciences and Biomedical Engineering, Indian Institute of Technology Indore, Khandwa, India

**Keywords:** SARS-CoV-2, nsp16/nsp10, molecular dynamics, PCA, MM-PBSA

## Abstract

Recently, a highly contagious novel coronavirus disease 2019 (COVID-19), caused by SARS-CoV-2, has emerged, posing a global threat to public health. Identifying a potential target and developing vaccines or antiviral drugs is an urgent demand in the absence of approved therapeutic agents. The 5′-capping mechanism of eukaryotic mRNA and some viruses such as coronaviruses (CoVs) are essential for maintaining the RNA stability and protein translation in the virus. SARS-CoV-2 encodes S-adenosyl-L-methionine (SAM) dependent methyltransferase (MTase) enzyme characterized by nsp16 (2′-O-MTase) for generating the capped structure. The present study highlights the binding mechanism of nsp16 and nsp10 to identify the role of nsp10 in MTase activity. Furthermore, we investigated the conformational dynamics and energetics behind the binding of SAM to nsp16 and nsp16/nsp10 heterodimer by employing molecular dynamics simulations in conjunction with the Molecular Mechanics Poisson-Boltzmann Surface Area (MM/PBSA) method. We observed from our simulations that the presence of nsp10 increases the favorable van der Waals and electrostatic interactions between SAM and nsp16. Thus, nsp10 acts as a stimulator for the strong binding of SAM to nsp16. The hydrophobic interactions were predominately identified for the nsp16-nsp10 interactions. Also, the stable hydrogen bonds between Ala83 (nsp16) and Tyr96 (nsp10), and between Gln87 (nsp16) and Leu45 (nsp10) play a vital role in the dimerization of nsp16 and nsp10. Besides, Computational Alanine Scanning (CAS) mutagenesis was performed, which revealed hotspot mutants, namely I40A, V104A, and R86A for the dimer association. Hence, the dimer interface of nsp16/nsp10 could also be a potential target in retarding the 2′-O-MTase activity in SARS-CoV-2. Overall, our study provides a comprehensive understanding of the dynamic and thermodynamic process of binding nsp16 and nsp10 that will contribute to the novel design of peptide inhibitors based on nsp16.

## Introduction

Coronaviruses (CoVs) are considered as an etiological agent for causing severe acute respiratory syndrome (SARS) in humans. In the past two decades, SARS-CoV and MERS-CoV (middle east respiratory syndrome coronavirus) were responsible for the epidemic in 2003 and 2012, respectively. Recently, in December 2019, the novel coronavirus (COVID-19) pandemic, caused by SARS-CoV-2, first broke out in Wuhan city of China and has been spreading worldwide (Bogoch et al., 2020; Guan et al., 2020; Lin X. et al., 2020). So far, ~42 million people worldwide are infected by SARS-CoV-2, including ~1.1 million deaths. In India, the COVID-19 tally has already crossed 7.7 million, including ~0.1 million deaths. However, neither prophylactic vaccines nor any direct antiviral drugs are available to effectively treat the human and animal coronavirus disease (Lu, 2020; Pillaiyar et al., 2020; Sheahan et al., 2020).

CoVs are single-stranded positive-sense RNA viruses (Eckerle et al., 2010; Fehr and Perlman, 2015) belonging to the family *Coronaviridae* and possess the largest genome (26.4–31.7 kb) (Woo et al., 2010). They are classified into four genera, namely *Alphacoronaviruses, Betacoronaviruses, Gammacoronaviruses*, and *Deltacoronaviruses* (King et al., 2011). CoVs can infect both humans and animals (Lun and Qu, 2004; Coleman and Frieman, 2014), and can cause diseases like Hepatitis and Pneumonitis in mouse (Weiss and Leibowitz, 2011) and neurologic & respiratory diseases in humans (Arabi et al., 2015; Huang et al., 2020). So far, seven distinctive strains of human coronaviruses (HCoV) have been disclosed that includes 229E and NL63 (*Alphacoronaviruses*), and OC43, HKU1, SARS-CoV (2002), MERS-CoV (2012), and SARS-CoV-2 (2019) (*Betacoronaviruses*) (Weiss and Navas-Martin, 2005; Zeng et al., 2018; Singh et al., 2020). SARS-CoV-2 shares a strong correlation with the bat CoV RaTG13 having a 96.2% genome sequence identity, suggesting its possible evolution from bat CoVs (Wu et al., 2020; Zhou P. et al., 2020). SARS-CoV-2 displays a sequence similarity of 80% with SARS-CoV, whereas only 50% with MERS-CoV (Lu et al., 2020; Wu et al., 2020; Zhou Y. et al., 2020). Currently, most of the therapeutic options that are available for controlling COVID-19 is based on the previous knowledge and information gathered from SARS-CoV and MERS-CoV.

Most viruses or eukaryotic cellular mRNA possess the 5′-end capping mechanism that plays a vital role in mRNA splicing, translation initiation, stability, and intracellular RNA transport (Furuichi and Shatkin, 2000). The capping of the 5′-end occurs through a sequential enzymatic process. This involves three enzymes, such as RNA guanylyltransferase (GTase), RNA triphosphatase (TPase), and RNA guanine-N7-methyltransferase (N7-MTase). This generates a cap-0 structure (m7GpppN). It is further methylated at the 2′-O position of mRNA by 2′-O- methyltransferase (2′-O-MTase) and generates the cap-1 (m7GpppNm) and cap-2 (m7GpppNmNm) structures. This mimicking of the eukaryotic mRNA capping mechanism ultimately helps the virus evade the host innate immune system (Furuichi and Shatkin, 2000; Wang et al., 2015). Both the MTase uses S-adenosyl-L-methionine (SAM or AdoMet) as a donor of methyl and gives a by-product, S-adenosyl-L-homocysteine (SAH or AdoHcy).

The genome of SARS-CoV-2 is comprised of 10 ORFs (Open Reading Frame). ORF1ab encodes the replicase polyprotein 1ab (PP1ab), which gets cleaved by the two viral proteases, PL^pro^ (papain-like protease) and 3CL^pro^ (3-C like protease) at the N-terminus and C-terminus, respectively, to form all the 16 non-structural proteins (nsp1, nsp2, nsp3 by PL^pro^, and nsp4-nsp16 by 3CL^pro^). The remaining ORFs are associated with encoding structural proteins, such as spike (S), envelope (E), membrane (M), and nucleocapsid (N) proteins, as well as other accessory proteins (Harcourt et al., 2004; Yang et al., 2005; Chen et al., 2020; Wu et al., 2020). Previous structural and biochemical characterization studies on SARS-CoV (2002) showed that in the nsp10-nsp16/SAM complex, nsp10 enhances the methylase activity of nsp16 by increasing the stability of the SAM-binding pocket (Chen et al., 2011; Wang et al., 2015).

**Figure 1:**
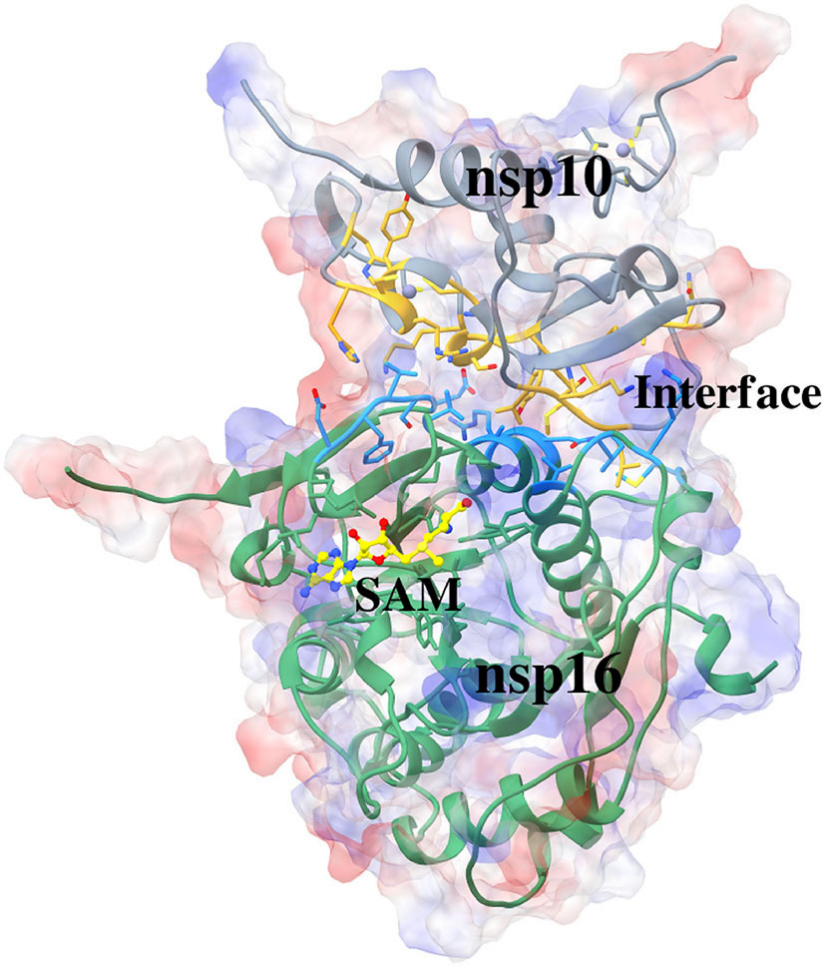
Crystal structure of SARS-CoV-2 nsp10-nsp16 complexed with cofactor S-Adenosyl Methionine (SAM) (PDB: 6W4H). The nsp10, interacting part of nsp10, interacting part of nsp16, nsp16, and the cofactor SAM in a complex is shown in color teal, gold, dark sky blue, light green, and yellow, respectively. The surface represents an electrostatic surface.

The present study involves analyzing protein-protein interactions in the heterodimeric structure formed by nsp10 and nsp16 of SARS-CoV-2. The recently solved crystal structure of SARS-CoV-2 nsp16/nsp10 heterodimer bound to SAM (PDB: 6W4H) (Minasov et al., 2020) is used in the current study. The three-dimensional structure of the complex is shown in Figure 1. Here, we have used the standard molecular dynamics (MD) simulations in conjunction with the molecular-mechanics Poisson-Boltzmann surface area (MM-PBSA) method to identify the critical residues involved in the complex formation. Further, we conducted MD simulations of nsp16/SAM, elucidating the importance of nsp10 in the binding of SAM to nsp16. The detailed structural analysis and inter-molecular interactions reveal the interface of nsp16/ns10, which provides a distinctive feature for coronaviruses, can be exploited as an attractive target in developing specific antiviral drugs for controlling COVID-19. MD simulations were conducted to elucidate the role of nsp10 in the interaction of SAM to nsp16. Also, the conformational changes in nsp10 and nsp16 due to their association were investigated.

## Materials and Methods

We retrieved the experimental coordinate of the SARS-CoV-2 nsp16/nsp10 complex from Protein Data Bank (PDB), which was crystallized at 1.8 Å (PDB ID 6W4H) (Minasov et al., 2020). The complex structure includes S-adenosyl-L-methionine (SAM). The protonation states were determined using PROPKA 3.1 (Olsson et al., 2011), and the corresponding residues were modified accordingly. The following three systems were simulated for the current study: complex (nsp16/nsp10/SAM), nsp16_SAM_ (nsp16/SAM), and nsp10_apo_ (apo nsp10). The starting configurations of nsp16_SAM_ and nsp10_apo_ were constructed manually from nsp16/nsp10/SAM (PDB: 6W4H).

Missing hydrogens in the crystal structure were built using the *Leap* module of AmberTools19 (Case et al., 2018), and a proper amount of Na^+^ was added to neutralize the system. All the systems were solvated in a periodic octahedron TIP3P water box (Price and Brooks, 2004) with a 10 Å buffering distance from all directions. The proteins (nsp10 and nsp16) were assigned the Amber ff14SB force field parameters (Maier et al., 2015) while the cofactor SAM was modeled using the updated Generalized Amber Force Field (GAFF2) (Wang et al., 2004) and AM1-bcc (Jakalian et al., 2002) charges. The bond lengths having hydrogen atoms were kept fixed using the SHAKE algorithm (Kräutler et al., 2001), which allowed 2 fs time-step for MD simulations. The Particle-Mesh Ewald (PME) summation (Darden et al., 1993) scheme was employed to compute the long-range interactions. The non-bonded cut-off was set to 10 Å.

Firstly, the solvated systems were subjected to an energy minimization using 500 steps of steepest descent, followed by another 500 steps of the conjugant gradient algorithm. During the minimization, the solute atoms were kept fixed with a restraint force of 2.0 kcal mol^−1^Å^−2^. The second stage of minimization was carried out without any restraint force. After the minimization, each system was gradually heated from 0 to 300 K in the NVT ensemble, and the solute atoms were restrained with force constant of 2.0 kcal mol^−1^Å^−2^. The temperature was maintained using a Langevin thermostat (Pastor et al., 1988; Loncharich et al., 1992). Subsequently, each system was simulated for 50 ps at 300 K at a constant pressure of 1 atm using the Berendsen barostat (Berendsen et al., 1984). In this stage also, the same restraint force was applied to the solute atoms. Before the production run, we equilibrated each system by conducting 1 ns MD simulation in the NPT ensemble without restraining the system. Finally, the production simulation was carried out for 1 μs using the *pmemd*.*cuda* module of AMBER18. Overall, 100,000 snapshots were generated and used for the analysis using the *Cpptraj* module (Roe and Cheatham, 2013) of AMBER18 (Case et al., 2018).

To study the effect of salt concentration on the binding, we simulated the complex system at 300 K for 1 μs at two different salt concentrations (0.15 and 0.25 M). The desired salt concentration was achieved by adding an appropriate number of monovalent ions (Na^+^ and Cl^−^) obtained with the protocol suggested by Machado and Pantano (2020). Similarly, we have also investigated the effect of temperature on the binding by conducting MD simulation of the complex at a relatively elevated temperature of 310 K.

### Trajectory Analysis

All analyses, including root-mean-square deviations (RMSDs), root-mean-square fluctuations (RMSFs), radius of gyration (R_g_), solvent accessible surface area (SASA), etc. were performed using the *Cpptraj* module (Roe and Cheatham, 2013) of AMBER18. A distance of ≤ 3.5 Å and an angle cut-off of ≥120° were used for hydrogen bond calculations. The same criterion was used in earlier studies (Jeffrey, 1997; Hu et al., 2016; Shi et al., 2018; Sanachai et al., 2020).

### Principal Component Analysis

One of the widely used unsupervised data reduction schemes is principal component analysis (PCA) (Ichiye and Karplus, 1991). First, a covariance matrix (C) is constructed from the atomic fluctuations of C_α_-atoms of each residue (Amadei et al., 1996). The elements *C*_ij_ of the covariance matrix *C* are defines as

(1) $$\begin{array}{l} C_{ij}=<\left(\left(x_{i}-<x_{i}>\right)\left(x_{j}-<x_{j}>\right)\right)> \end{array}$$

where *x*_*i*_ and *x*_*j*_ denotes the instant coordinates of the *i*th or *j*th atoms, while < *x*_*i*_ > and < *x*_*j*_ > denote the mean of *i*th or *j*th atoms over the ensemble. The diagonalization of the covariance matrix yields orthogonal eigenvectors and the corresponding eigenvalues. The resulting eigenvectors are termed as principal components (PCs) and indicate the direction of the movement, while the corresponding eigenvalue describes the amplitude of motions. We adopted the same methodology for PCA, as discussed in our earlier studies (Jonniya et al., 2019; Sk et al., 2020c).

The free energy landscape (FEL) was generated based on the following Equation (Frauenfelder et al., 1991):

(2) $$\begin{array}{l} G_{i}=-k_{B}T\text{ln}\left(\frac{N_{i}}{N_{m}}\right) \end{array}$$

where *k*_*B*_ represents the Boltzmann constant, and *T* is the absolute temperature. *N*_i_ is the population of the *i*th bin, and *N*_m_ is the population of the most populated bin. The 2-dimensional FEL was constructed using PC1 and PC2 as the reaction coordinate.

### Residual Network Analysis of Protein

The residual network analysis approach is widely used to explore the viral fitness and resistance development of protein structure. The network analysis of protein structures (NAPS) (Chakrabarty et al., 2019) server (http://bioinf.iiit.ac.in/NAPS/) was used to identify key residue interactions in the residual network and the network-based hydrophobic contacts from the simulation trajectories. Here, we used the protein-protein complex option, followed by the C_α_ network type and unweighted edge weight. An edge represented the distance between a pair of C_α_ atoms within the lower and upper thresholds (default upper threshold = 7 Å; lower threshold = 0 Å). We considered the default parameters for the long-range interaction networks and minimum residue separation of 1. The hydrophobicity indices of 20 amino acids range from −4.5 to 4.5 (Kyte and Doolittle, 1982). These values were colored in gradients of red to show the hydrophobic residues, while the gradients of blue were used to display the hydrophilic residues.

### Binding Free Energy and Alanine Scanning

The interaction energy between nsp10 and nsp16, and between the cofactor SAM and nsp16 in its both monomer and the complex were computed using the molecular mechanics Poisson-Boltzmann surface area (MM-PBSA) methodology (Kollman et al., 2000; Wang et al., 2006; Kar et al., 2007a,b, 2011, 2013; Hou et al., 2011). MMPBSA.py script available in the AmberTools19 was used for the analysis. Details of the MM-PBSA protocol were provided in our previous studies (Kar and Knecht, 2012a,b,c,d; Kar et al., 2013; Jonniya et al., 2019; Jonniya and Kar, 2020; Roy et al., 2020; Sk et al., 2020a,d), and the same protocol was adopted here.

The binding free energy is estimated by using the following equations of the MM-PBSA scheme:

(3) $$\begin{array}{l} \Delta G_{\text{bind}}=\Delta H-T\Delta S\approx \Delta E_{\text{internal}}+\Delta G_{\text{solv}}-T\Delta S \end{array}$$

(4) $$\begin{array}{l} \Delta E_{\text{internal}}=\Delta E_{\text{covalent}}+\Delta E_{\text{elec}}+\Delta E_{\text{vdW}} \end{array}$$

(5) $$\begin{array}{l} \Delta G_{\text{solv}}=\Delta G_{\text{pol}}+\Delta G_{\text{np}} \end{array}$$

where Δ*E*_internal_, Δ*G*_solv_, and *TΔS* represent the total internal energy, desolvation free energy, and conformational entropy, respectively. Further, the internal energy is composed of Δ*E*_covalent_ (bond, dihedral, and angle), Δ*E*_elec_ (electrostatic) and Δ*E*_vdW_ (van der Waals) and the desolvation free energy is composed of polar (Δ*G*_pol_) and non-polar solvation energies (Δ*G*_np_). Here, Δ*G*_pol_ was estimated from the Poisson-Boltzmann (PB) equation. The dielectric constant of the solute and solvent was set to 1.0 and 80.0, respectively. Δ*G*_np_ was estimated from the following Equation (6),

(6) $$\begin{array}{l} \Delta G_{np}=\gamma \left(SASA\right)+b \end{array}$$

Here, *SASA* represented the solvent-accessible surface area and was estimated using the LCPO (linear combination of pairwise overlap) algorithm (Weiser et al., 1999) with a probe radius of 1.4 Å. The surface tension coefficient, γ and offset (b) value were set to 0.00542 kcal.mol^−1^.Å^−2^ and 0.92 kcal.mol^−1^, respectively (Gohlke et al., 2003). For the MM-PBSA calculation, 2000 snapshots selected from the last 300 ns trajectory with a frequency of 15 ps were employed.

The configurational entropy was calculated using the normal mode analysis (NMA) method (Karplus and Kushick, 1981; Rempe and Jónsson, 1998; Xu et al., 2011), and ~300 snapshots were used in the calculation due to high computational cost. Each configuration was energy minimized in an implicit solvent (nmode_igb = 1) using a maximum of 50,000 steps, and a target root-mean-square gradient of 10^−4^ kcal mol^−1^Å^−1^ via *mmpbsa_py_nabnmode* (Hawkins et al., 1995, 1996; Kar and Knecht, 2012a,b,c; Kar et al., 2013). NMA was applied to calculate the vibrational entropy (*S*_vib_) using the following equation (Carlsson and Åqvist, 2006);

(7) $$S_{vib}=R[\frac{x}{\left(e^{x}-1\right)}-ln(1-e^{-x})];x=\frac{h\upsilon }{kT}$$

Where *R* denotes the universal gas constant, *k* is the Boltzmann constant, and *T* is the absolute temperature. The Planck constant and the vibrational frequency are denoted as *h* and ν, respectively.

Further, the decomposition of the total binding free energy at the residue level was conducted by using the Molecular Mechanics Generalized Born Surface Area (MM-GBSA) scheme (Kar and Knecht, 2012a,b,c; Kar et al., 2013; Jonniya and Kar, 2020) as per the following equation.

(8) $$\begin{array}{l} \Delta G_{\text{residue}}=\Delta E_{\text{vdW}}+\Delta E_{\text{ele}}+\Delta vG_{\text{pol}}+\Delta G_{\text{np}} \end{array}$$

The total contribution from each residue can also be defined as the sum of van der Waals (Δ*E*_vdW_), electrostatic (Δ*E*_ele_), polar (Δ*G*_pol_), and non-polar (Δ*G*_np_) terms. This method was proposed by Gohlke et al. (2003).

Finally, the Computational Alanine Scanning (CAS) was performed for some essential residues. This method yields the energy difference between the wild type and mutant (alanine) variants.

(9) $$\begin{array}{l} \Delta \Delta G_{\text{bind}}=\Delta G_{\text{mutant}}-\Delta G_{\text{wild}} \end{array}$$

The basic principle involves in AS (alanine scanning) is the substitution of a residue with alanine that has an impact on the side-chain beyond C_β_ and not in the main-chain. *In vitro*, AS has been proven an advantageous mutagenesis method in finding hotspot residues in protein-protein interfaces. CAS is an excellent alternative approach to the *in vitro* experimental alanine scanning (Massova and Kollman, 1999; Moreira et al., 2007). Therefore, in this study, CAS was applied to illustrate further the importance of specific residues except alanine mentioned in the binding decomposition free energy of nsp16 of COVID-19.

## Results and Discussion

To explore the mechanism underlying the dimerization of nsp16 and nsp10 as well as the preferential binding of the cofactor SAM to the nsp16/nsp10 heterodimer compared to nsp16 (nsp16_SAM_), a conformational free energy landscape (FEL) and binding free energy calculations were performed using the MD/MM-PBSA scheme.

### Overall Structural Dynamic Features

**Figure 2:**
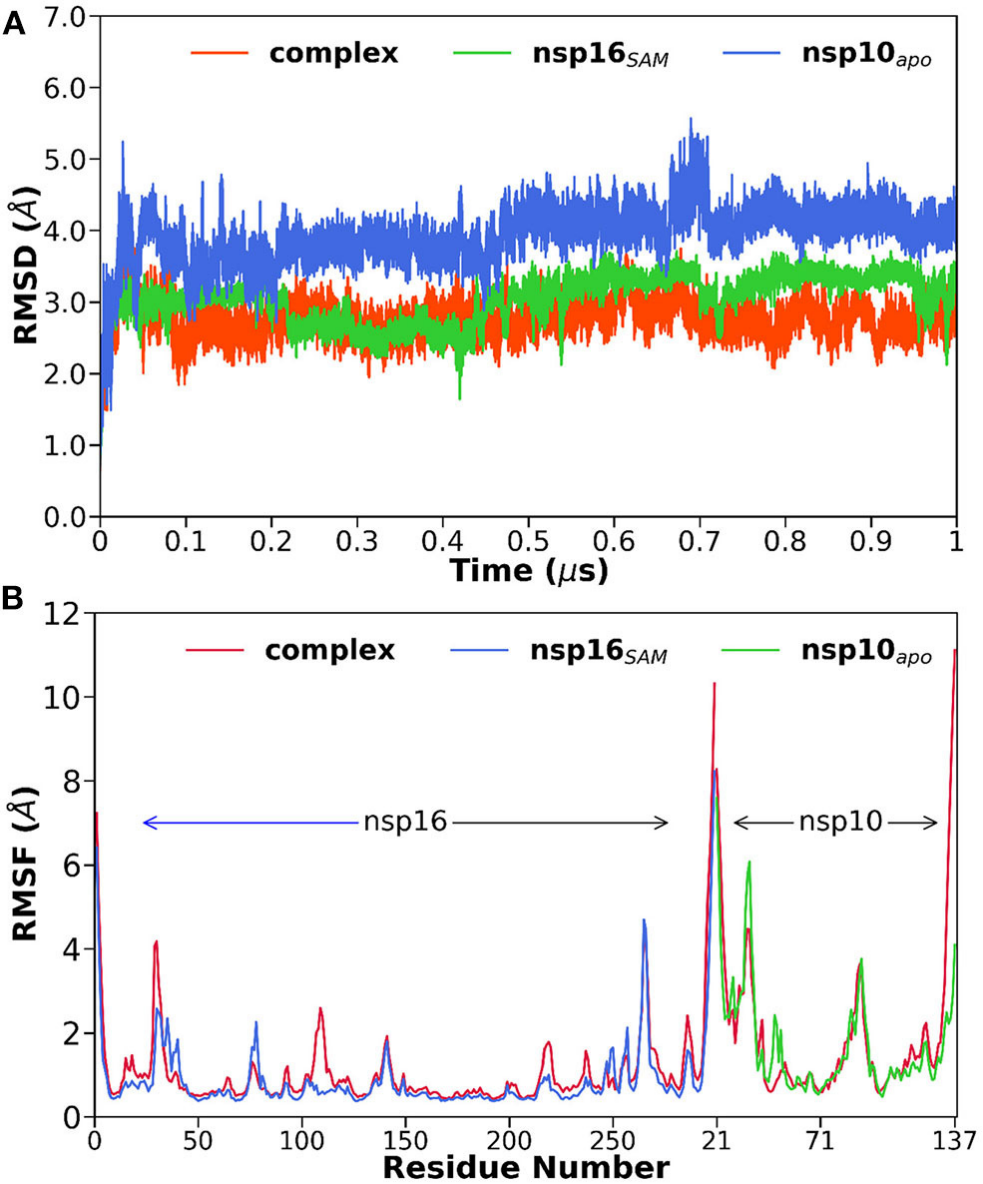
**(A)** The root-mean-square deviations (RMSDs) of the backbone atoms relative to their initial coordinates as a function of simulation time, **(B)** the root-mean-square fluctuations (RMSFs) of C_α_ atoms for each residue in the complex and monomers of nsp16_SAM_ and nsp10_apo_.

To explore the thermodynamic stability of each system and to ensure the rationality of the sampling method, we monitored the structural and energetic properties during the entire 1 μs production simulation. The time evolution of the root-mean-square deviations (RMSDs) of the protein backbone atoms, which reflects the stability of the system, were calculated relative to the initial configuration and shown in Figure 2A. It is worth mentioning here that a stable RMSD does not always provide stable energy profiles; hence we have also verified the potential and total energy of each system from the respective MD trajectory (data not shown).

The RMSD plots indicated that all the studied systems reached equilibrium after ~100 ns. The average RMSD value varies between 2.8 and 4.0 Å for all systems (see Table 1). The highest and lowest deviations were obtained for nsp10_apo_ and complex, respectively. An intermediate RMSD value of 3.1 Å was obtained for nsp16_SAM_. Overall, the nsp16/nsp10/SAM complex showed more stability throughout the simulations than nsp16_SAM_ or nsp10_apo_. This suggests that the dimerization leads to the overall stabilization of the complex.

**Table 1:** The average value of protein backbone RMSD and solvent-accessible surface area (SASA) obtained from MD simulations.

**System**	**RMSD (Å)**	**SASA (Å^**2**^)**
Complex	2.8 (0.1)	18928.1 (103.3)
nsp16_SAM_	3.1 (0.3)	13467.3 (140.2)
nsp10_apo_	4.0 (0.2)	6663.8 (145.7)

*Standard deviations are given in parentheses*.

Moreover, we also calculated the temporal RMSDs of heavy atoms of the cofactor SAM (see Supplementary Figure 1A) and the backbone atoms of residues within 5 Å around SAM in the binding pocket (see Supplementary Figure 1B). It is evident from Supplementary Figure 1B that the RMSD values of SAM and the binding pocket for the complex were stable up to 850 ns of the simulation. After that, in the last 150 ns, a sudden increase in RMSD was observed. Overall, the average RMSD value was 0.7 Å and 0.8 Å for SAM and the binding pocket, respectively (see Supplementary Table 1). It indicates that SAM binds strongly onto the cofactor binding cavity of the heterodimer nsp16/nsp10. However, in the case of nsp16_SAM_, the RMSD values of SAM and the binding cavity were relatively stable up to 100 ns. After that, the RMSD value increased by ~3 times compared to the complex system (see Supplementary Table 1 and Supplementary Figure 1). These high values of RMSDs suggest that SAM does not bind to nsp16 monomer (nsp16_SAM_), which is in agreement with the experimental study by Chen and coworkers for SARS-CoV (Chen et al., 2011).

Additionally, three loops, namely 71–79, 100–108, and 130–148, which comprises the SAM binding pocket as shown in the crystal structure, were also analyzed and found to be stable for both complex and nsp16_SAM_ (see Supplementary Figures 2A–C). However, the cap-binding groove of nsp16 in SARS-CoV, as well as SARS-CoV-2, is mainly composed of two flexible loops, comprised of residues 26–38 and 130–148, while in the case of flavivirus, the NS5 MTase was relatively stable with α-helices (A1, A2, and half of αD) along the cap-binding groove (Egloff et al., 2002). Among the two loops involved in the cap-binding, only the loop 26–38 exhibited differences in the complex and nsp16_SAM_ system, as revealed from our simulations. The 26–38 loop is located near the interface of nsp16 and nsp10. On the other hand, the 130–148 loop is found near the SAM binding pocket, away from the nsp16/nsp10 interface. As shown in Supplementary Table 1, the average RMSD value of this loop was higher in nsp16_SAM_ (3.1 Å) compared to the complex (1.4 Å). The time evolution of RMSD of the 26–38 loop and the corresponding potential mean force (PMF) were displayed in Supplementary Figures 3A,B. From Supplementary Figure 3A, it was observed that in the case of nsp16_SAM_, the RMSD value of the loop 26–38 increased during the initial 300 ns and remained stable thereafter. On the other hand, in the complex case, the RMSD of the loop was found to increase during the initial 300 ns and gradually decreased up to 600 ns of the simulation and finally reached equilibrium. We computed the potential of mean force (PMF), taking the loop's RMSD as a reaction coordinate and shown in Supplementary Figure 3B. The primary low energy structure of the loop was observed at ~3.2 Å for nsp16_SAM_. However, for the complex system, the global energy minimum was found at a relatively lower RMSD (1.4 Å). Besides, we detected two secondary minima (~1.9 Å, and ~2.8 Å) for the 26–38 loop in the complex system, and the energy barriers of the adjacent states were ~1.1 and 1.2 kcal/mol, respectively. Overall, our results suggest that the dimerization resulted in the loop rearrangement near the interface.

Next, we measured the root-mean-square fluctuations (RMSFs) of Cα atoms for all systems (see Figure 2B) to elucidate the effect of dimerization on the residual flexibility. It is evident from Figure 2B that all three systems displayed a similar trend in RMSF patterns. However, we found some dynamic fluctuations in different loop regions, including the N- and C-terminals. A relatively large fluctuation was observed for various loop regions located at residues 26–38, 74–80, 104–115, and 130–148. The loop regions 26–38 and 104–115, which contribute to the interface of the heterodimer, showed higher fluctuations in complex compared to nsp16SAM, which suggests that the dimerization leads to the loop rearrangements. In contrast, the 74–80 loop region, located near SAM binding pocket, stabilized after the heterodimerization. However, the cap-binding groove of the 130–148 loop region exhibited no differences in both complex and apo forms, which agrees with the RMSD analysis (Supplementary Figure 2C). Overall, it depicts the binding of nsp10 to nsp16 favors and stabilizes the binding pocket of SAM, leading to its high activity.

Finally, we also measured the solvent-accessible surface area (SASA) of all systems and reported in Table 1. The average SASA values were found to be 18928.1, 13467.3, and 6663.8 Å^2^, for the complex, nsp16_SAM_, and nsp10_apo_, respectively. This suggested that the dimerization of nsp16 and nsp10 covered ~1,203 Å^2^ surface area in total, indicating a very stable interaction. Our simulation results also favor an experimental study that showed the value of the solvent-exposed surface area for the heterodimer complex of nsp16/nsp10 as 19710 Å^2^ (Rosas-Lemus et al., 2020).

### Free Energy Landscape (FEL) of SAM Binding

**Figure 3:**
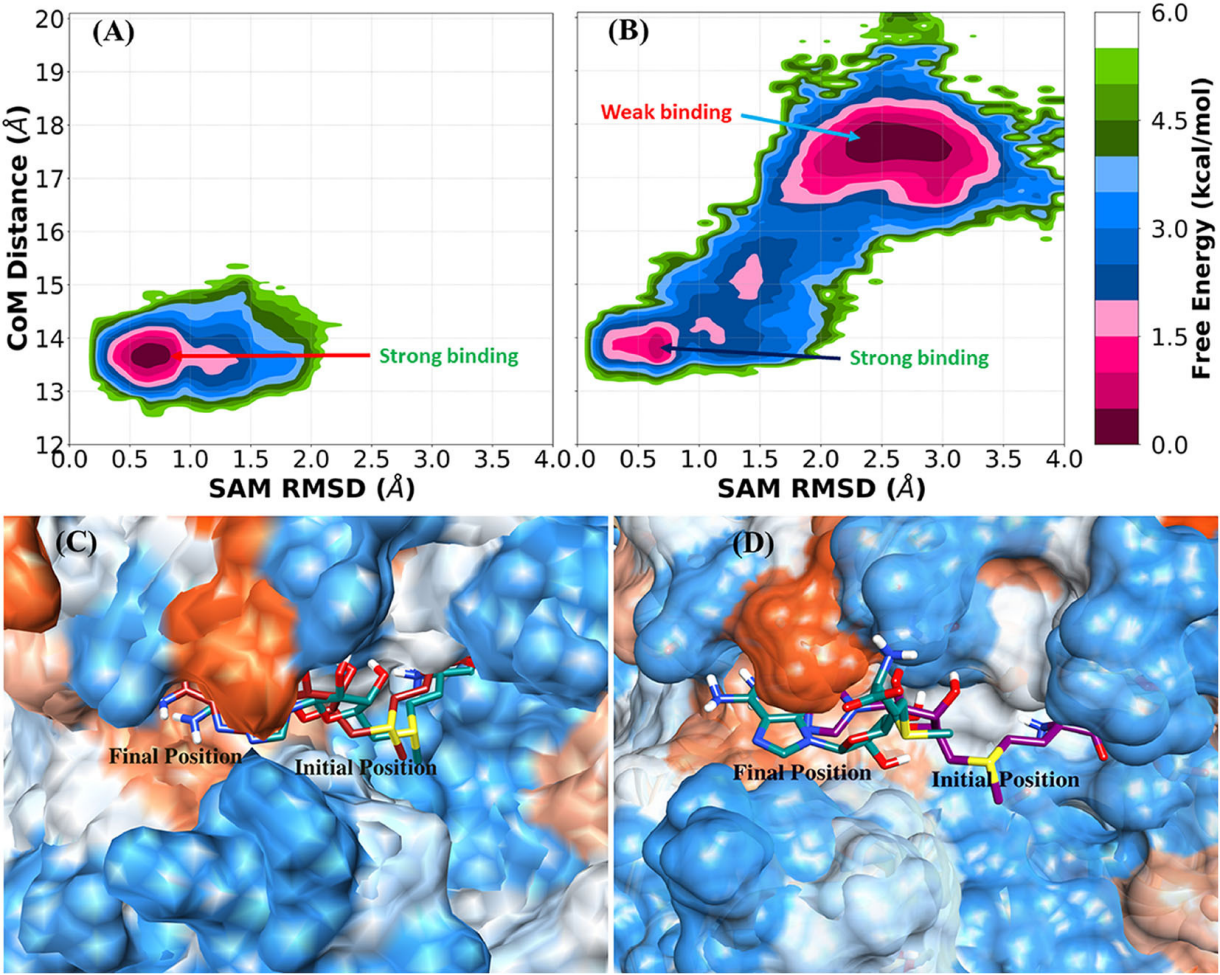
Free energy landscape (FEL) and representative SAM position in the complex (nsp16/nsp10/SAM) and nsp16_SAM_ (nsp16/SAM). **(A)** FEL of SAM bound with nsp16 in the complex, **(B)** FEL of SAM bound with monomer nsp16_SAM_. Representative structures of **(C)** SAM with nsp16 in the complex and **(D)** SAM with monomer nsp16_SAM_.

The RMSD profile of SAM and the binding pocket (Supplementary Figure 1) for the complex and nsp16_SAM_ systems suggested that SAM binds more strongly to the heterodimer nsp16/nsp10 than the monomer nsp16. The 2-dimensional free energy landscape (FEL) was generated for the complex and nsp16_SAM_ systems to explore the strong and weakly bound states of the SAM molecule. The RMSD of heavy atoms of SAM and the center of mass (CoM) distance between SAM and nsp16, which characterizes the displacement of SAM from the substrate-binding pocket of nsp16, were used as reaction coordinates for the construction of FEL. The value of a CoM distance <14.5 Å denotes the strong binding of SAM toward nsp16. The two-dimensional FEL of the complex (nsp16/nsp10/SAM) and monomer (nsp16_SAM_) systems were shown in Figures 3A,B, and the corresponding positions of SAM in the binding cavity were depicted in Figures 3C,D. FEL of the complex and monomer systems suggested that the conformational state of SAM in two systems prefer disparate configurations. In the case of the heterodimeric complex system, the underlying FEL was characterized by a single global minimum (see Figure 3A), which corresponds to the strongly bound state of SAM. On the other hand, in the case of nsp16_SAM_, the global free energy minimum was obtained at a CoM distance of ~17.5 Å, which corresponds to a weakly or unbound state of SAM. The secondary minimum was detected at a CoM distance of ~13.5 Å, which corresponds to a strongly bound state of SAM to nsp16_SAM_. Overall, our results suggested that although nsp16 monomer (nsp16_SAM_) favored two binding states, the weakly bound or unbound state is energetically more favorable than the strongly bound state. These results illustrated a stronger binding of SAM with the nsp16/nsp10 heterodimer than nsp16 alone (nsp16_SAM_), which agrees with the experimental results obtained for SARS-CoV (Chen et al., 2011).

### Principal Component Analysis of nsp16/nsp10

The principal component analysis (PCA) was carried out for both nsp16 and nsp10 when they are in complex and monomeric states. FELs of sub-units nsp16 (nsp16_complex_) and nsp10 (nsp10_complex_) in the complex were compared with the corresponding monomer simulations (nsp16_SAM_ and nsp10_apo_). Each eigenvector and eigenvalue was plotted in the decreasing order, as shown in Supplementary Figure 4. For all cases, the first few eigenvectors describe the collective motion of local fluctuations. Comparing the four systems indicated that the first few PCs that describe the properties of movements were not the same. The first two eigenvectors encapsulated for 42, 67, 46, and 70% of overall movements in nsp16_complex_ (nsp16 in complex), nsp10_complex_ (nsp10 in complex), nsp16_SAM_, and nsp10_apo_, respectively. Similarly, the first ten eigenvectors accounted for 80–90% of the total motions in all four systems.

**Figure 4:**
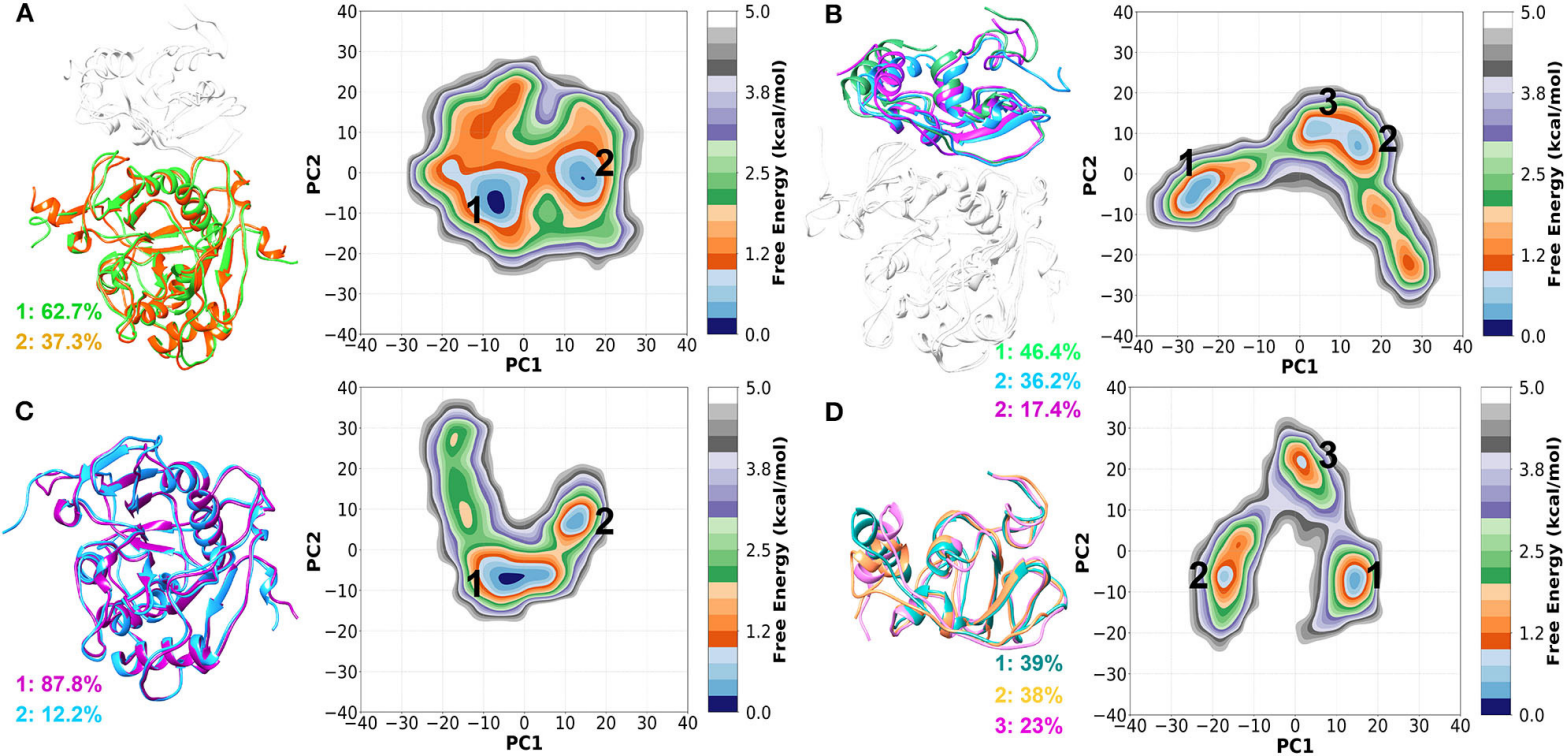
Two-dimensional FEL generated by projecting the first two principal components, PC1 and PC2 for **(A)** nsp16_complex_ (nsp16 in nsp16/nsp10/SAM), **(B)** nsp10_complex_ (nsp10 in nsp16/nsp10/SAM), **(C)** nsp16_SAM_ (nsp16/SAM), and **(D)** nsp10_apo_. The representative structures are shown on the left panel.

Next, we constructed the 2-dimensional FEL at 300 K for each system using the first two principal components (PC1 and PC2) as reaction coordinates and shown in Figures 4A–D. The sampling space of four systems was different, as evident from Figure 4. The conformational sampling space of nsp16_complex_ (nsp16 in nsp16/nsp10/SAM), depicted in Figure 4A, was found to be restricted compared to the other three systems, which sampled more expansive conformational space. From Figure 4A, we observed a global minimum of 62.7% occupancy and a secondary minimum of 37.3% occupancy, which suggested that the nsp16_complex_ system is more stabilized in the presence of nsp10. In the monomeric form of nsp16_SAM_, a wider conformational basin was sampled (see Figure 4C), and the energy barriers between adjacent minima were ~1.5 kcal/mol. On the other hand, nsp10 sampled more phase space than nsp16 in the complex as well in its monomer apo form, as shown in Figures 4B,D. However, nsp10_apo_ has three distinct conformations with an energy barrier of 4.0 kcal/mol suggesting structural disparity in both nsp10_complex_ and nsp10_apo_ as compared to nsp16.

### Residual Network Analysis

**Figure 5:**
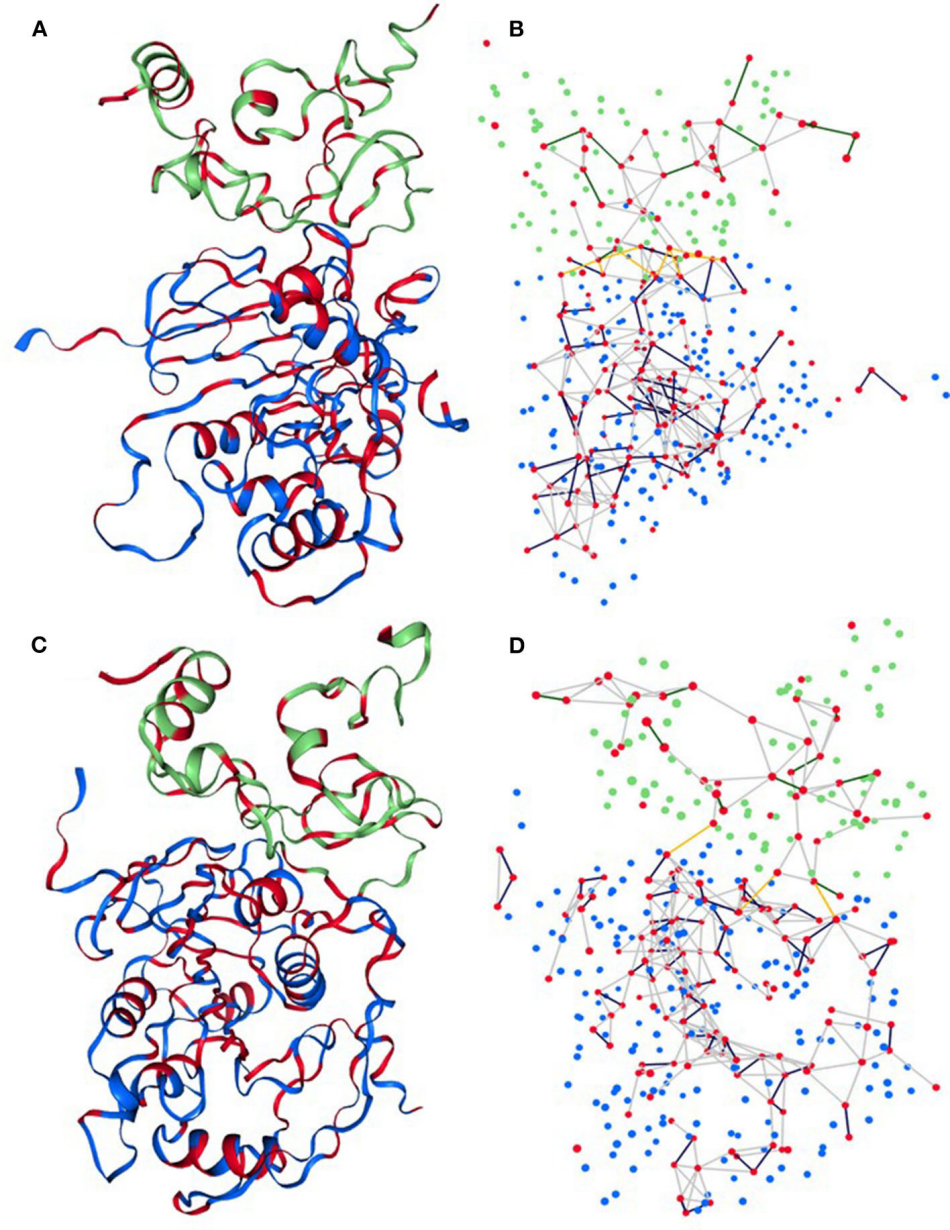
Sub-network representation of network 3D view of **(A,B)** initial structure and **(C,D)** final structure of the complex. Each residual C_α_ atom is represented by a sphere where the red sphere indicates hydrophobic residues. Blue and green color spheres correspond to residues of nsp16 and nsp10, respectively. The yellow line edges represent the contact network of hydrophobic residues between nsp16 and nsp10.

The Network Analysis of Protein Structure (NAPS) server provided a visual examination of sub-network based on the physicochemical properties of the protein residues to find more details about the interaction between nsp16 and nsp10 (see Figure 5). Herein, we explored the 3D interaction network of hydrophobic residues of nsp16 and nsp10. The results showed that the number of hydrophobic interaction networks between nsp16 and nsp10 was initially very high, and after 100 ns, the number of networks reduced to ~2–3. The hydrophobic interaction networks, such as (V104, **A71**) and (P80, **V42**), were found as strong and stable contacts throughout the simulation time (see Supplementary Table 2). We also calculated the total number of interactions and noticed that the number of interaction networks was more or less conserved throughout the first 500 ns (see Supplementary Figure 5). In general, it can be concluded from our analyses that nsp16 and nsp10 interact with a very high affinity.

### Binding Free Energy of SAM Bound to nsp16/nsp10 Heterodimer and nsp16 Alone

The differences in binding affinity of the SAM molecule toward nsp16 alone (nsp16_SAM_) and nsp16/nsp10 heterodimer (complex) were calculated by utilizing the MM-PBSA scheme. The MM-PBSA scheme provides various components contributing to the total binding energy (Δ*G*_bind_), such as van der Waals interactions (Δ*E*_vdW_), electrostatic interactions (Δ*E*_ele_), polar solvation energy (Δ*G*_pol_), non-polar solvation free energy (Δ*G*_np_), and configurational entropy (*T*Δ*S*). All these components of the binding free energy of SAM to nsp16_SAM_ or nsp16/nsp10 (complex) were shown in Table 2. The binding free energy of SAM to the heterodimer nsp16/nsp10 was estimated as −6.8 (± 0.9) kcal/mol, which agrees with the experimental result (−7.4 ± 0.1 kcal/mol) (Lin S. et al., 2020). On the other hand, SAM was found to bind very weakly (Δ*G*_bind_ = −0.6 ± 0.8 kcal/mol) to nsp16 monomer (nsp16_SAM_), as evident from Table 2. This is consistent with Figures 3A,B. A similar mode of SAM binding was observed for SARS-CoV nsp16/nsp10 or nsp16 alone. For both cases, the intermolecular van der Waals interactions (Δ*E*_vdW_), electrostatic interactions (Δ*E*_ele_), and non-polar solvation free energy (Δ*G*_np_) favored the binding of SAM. In contrast, polar solvation energy (Δ*G*_pol_) and entropy (*T*Δ*S*) disfavored the complexation. The electrostatic interaction energy was more favorable than the van der Waals interactions for both cases. In nsp16_SAM_, although Δ*E*_ele_ was much more favorable (−76.8 kcal/mol) than Δ*E*_vdW_ (−28.6 kcal/mol). However, the disfavorable polar solvation energy (Δ*G*_pol_ = 84.7 kcal/mol) overcompensated the intermolecular electrostatic interaction energy. Hence, the overall polar contribution (Δ*E*_ele_ + Δ*G*_pol_) was found to be unfavorable (7.9 kcal/mol) to the SAM binding. In contrast, the overall non-polar contribution (Δ*E*_vdW_ + Δ*G*_np_) was found to be −32.1 kcal/mol. It implies that hydrophobic interactions drive the binding. In the case of the complex system (nsp16/nsp10/SAM), the binding affinity of SAM to nsp16 increases, as evident from Table 2. Here, the electrostatic interactions energy (Δ*E*_ele_ = −181.1 kcal/mol) was more favorable than the van der Waals energy (Δ*E*_vdW_ = −40.4 kcal/mol). The total polar contributions (Δ*E*_ele_ + Δ*G*_pol_) value was found to be favorable (−2.1 kcal/mol) to the binding of SAM, which is in contrast to what had been observed for nsp16_SAM_. Further, the overall polar energy was less favorable than the net non-polar contribution (Δ*E*_vdW_ + Δ*G*_np_ = −44.5 kcal/mol). Hence, in the complex, the main force behind the binding of SAM to nsp16 is hydrophobic interactions. Overall, the binding free energy analysis showed that the binding affinity of the SAM molecule to nsp16 increases with the presence of nsp10. This implies that nsp10 acts as a stimulator to bind SAM to nsp16/nsp10 of SARS-CoV-2, which agrees with the experiment (Viswanathan et al., 2020). Wang and coworkers obtained a similar result for SARS-CoV and MERS-CoV (Wang et al., 2015).

**Table 2:** Energetic components of the total binding free energy of SAM bound to the heterodimer nsp16/nsp10 (complex), and nsp16 alone (nsp16_SAM_).

**System**	**ΔE_vdW_**	**ΔE_elec_**	**ΔG_pol_**	**ΔG_np_**	**ΔH**	**Δ−TS**	**ΔG_bind_**
Complex	−40.4 (0.1)	−181.1 (0.5)	179.0 (0.4)	−4.1 (0.0)	−46.6 (0.2)	39.8 (0.9)	−6.8 (0.9)
nsp16_SAM_	−28.6 (0.1)	−76.8 (0.6)	84.7 (0.6)	−3.5 (0.0)	−24.2 (0.1)	23.6 (0.8)	−0.6 (0.8)

*All values are given in kcal/mol. The standard error of the mean is provided in parentheses*.

**Figure 6:**
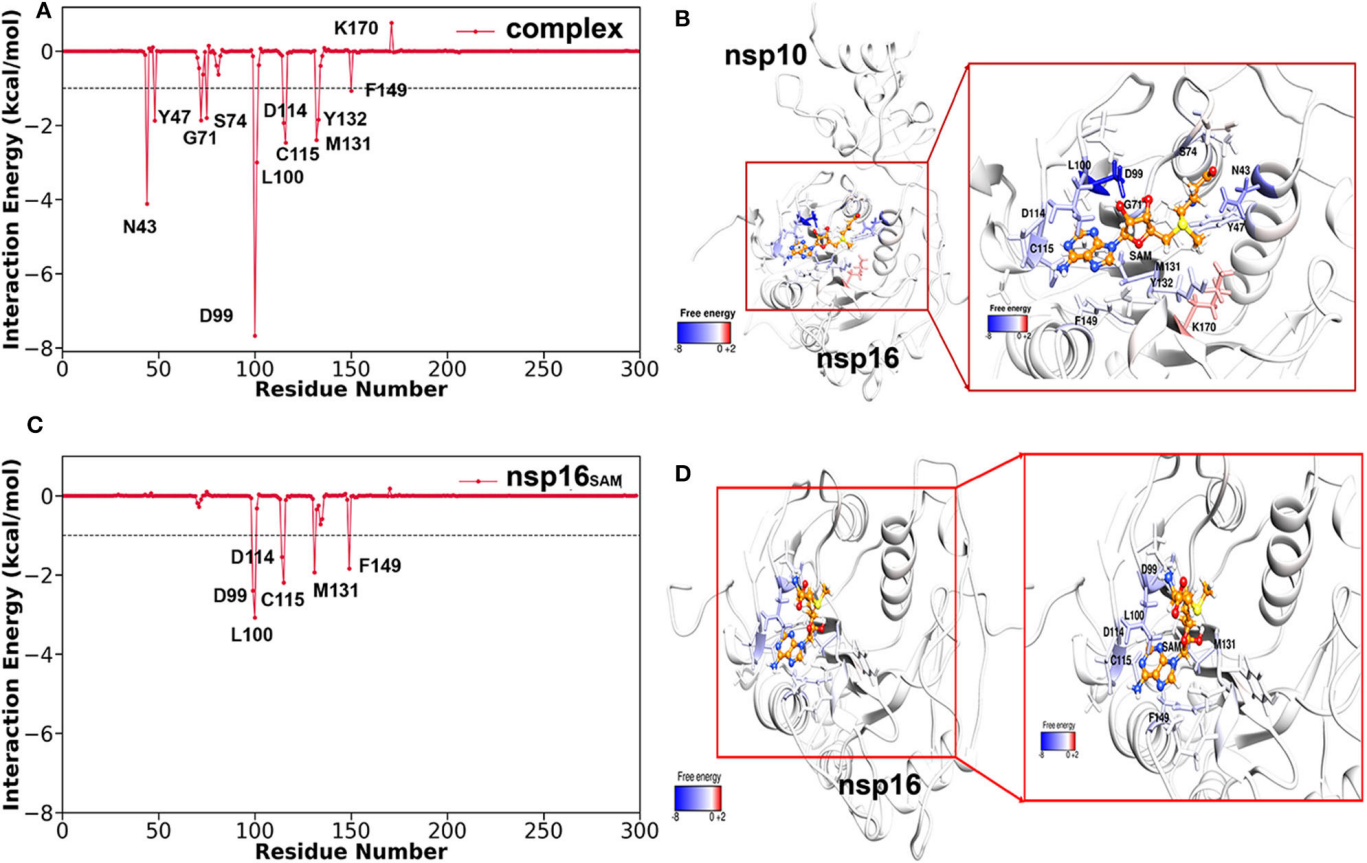
Per-residue decomposition free energy of nsp16 for the binding of SAM (in the presence of nsp10 and without nsp10) and respective binding pocket drawn from MD snapshots. **(A,B)** nsp16 with SAM in the presence of nsp10, **(C,D)** nsp16 with SAM without nsp10.

Further, to explore the significant residues of nsp16 in the binding of SAM and to evaluate the differences in the complex (nsp16/nsp10) and monomer (nsp16_SAM_), the per-residue decomposition of the binding free energy was performed using the MM-GBSA approach. All the residues having > 1.5 kcal/mol of energetic contributions were considered important and shown in Figure 6. As seen in Table 3, the number of amino acids contributing to binding with SAM is high in the complex as compared to nsp16_SAM_. Residues such as Leu100, Asp99, Cys115, Met131, Phe149, and Asp114 are common in cases of complex and nsp16_SAM_. Apart from these residues, additional residues, such as Asn43, Tyr47, Gly71, Tyr132, and Ser74, also played a significant role in the binding of SAM to the complex heterodimer. These results are consistent with the higher binding affinity of SAM in the complex than the nsp16 monomer. All these residues were also considered as SAM-engaging in an experimental study by Lin S. et al. (2020). It is worth noting here that all these SAM-interacting residues are conserved both in SARS-CoV and MERS-CoV, suggesting a conserved binding mode of SAM in these viruses. The conserved SAM-binding mechanism to nsp16/nsp10 also opens the possibility of developing a pan-CoV inhibitor by targeting this SAM-binding pocket.

**Table 3:** Per-residue decomposition of the total binding free energy (kcal/mol) of SAM to the complex (nsp16/nsp10) and monomer (nsp16_SAM_) systems.

**Residue**	**T_vdW_**	**T_ele_**	**T_pol_**	**T_np_**	**T_back_**	**T_side_**	**T_total_**
**COMPLEX**							
Asp99	0.71	−29.08	20.84	−0.14	−0.73	−6.94	−7.67
Asn43	−0.01	−9.90	5.89	−0.12	−0.30	−3.84	−4.14
Leu100	−2.42	−0.19	−0.10	−0.28	−0.87	−2.12	−2.99
Cys115	−0.74	−2.01	0.33	−0.05	−1.21	−1.26	−2.47
Met131	−2.72	0.56	−0.12	−0.13	−0.47	−1.94	−2.41
Asp114	−0.30	−8.98	7.41	−0.07	−0.77	−1.17	−1.94
Tyr47	0.07	−5.46	3.56	−0.04	−0.01	−1.86	−1.87
Gly71	−1.35	−3.27	2.84	−0.08	−1.36	−0.50	−1.86
Tyr132	−2.15	−2.23	2.86	−0.32	−0.93	−0.91	−1.84
Ser74	−0.95	−4.31	3.54	−0.08	−0.45	−1.35	−1.80
Phe149	−1.20	0.12	0.12	−0.13	−0.16	−0.93	−1.09
**nsp16**_**SAM**_							
Leu100	−3.04	1.61	−1.17	−0.47	−0.18	−2.89	−3.07
Asp99	0.62	−19.83	16.98	−0.16	−0.15	−2.24	−2.39
Cys115	−0.70	−1.73	0.30	−0.06	−1.16	−1.03	−2.19
Met131	−1.72	−0.02	−0.05	−0.15	−0.55	−1.39	−1.94
Phe149	−1.86	−0.33	0.54	−0.18	−0.20	−1.63	−1.83
Asp114	−0.28	−7.47	6.32	−0.10	−0.73	−0.80	−1.53

*The van der Waals (T_vdW_), electrostatic interactions (T_ele_), polar (T_pol_), and non-polar solvation energy (T_np_) and the total residual energy (T_total_) are shown. T_back_ and T_side_ represent the backbone and side-chain parts, respectively*.

### Hydrogen Bond Interaction Between nsp16 and SAM

Next, we investigated the hydrogen bond (H-bond) interactions between SAM and nsp16 in the complex and apo forms (see Table 4). The % occupancy calculated from the respective MD trajectories reflected the stability of H-bonds. As seen in Table 4, residues with > 50% H-bond occupancy in the MD simulations were recognized for the complex (nsp16/nsp10) compared to monomer nsp16_SAM_. The residues, including Asp130, Tyr47, Gly71, and Asp99, showed more H-bond stability in the complex. Overall, a stable H-bond is formed between nitrogen and oxygen atoms of SAM with Asp130, Tyr47, Gly71, and Asp99 residues of nsp16, respectively. Hence, the identified significant residues like Asp130, Tyr47, Gly71, and Asp99 of nsp16 may aid in the development of SAM competitive inhibitors.

**Table 4:** Hydrogen bonds formed between SAM and receptor (nsp16/nsp10 or nsp16).

**Binding couples**		**Molecular dynamics**	
**Acceptor**	**Donor…H**	**Distance (Å)**	**Occupancy^a^ (%)**
**COMPLEX**			
Asp130@OD2	SAM@N.HN1	2.84	71.69
SAM@N	Tyr47@OH.HH	2.79	64.95
Gly71@O	SAM@N.HN2	2.86	55.16
Asp99@OD2	SAM@O2′.HO2′	2.62	53.90
Asp99@OD1	SAM@O3′.HO3′	2.66	53.80
Asp99@OD2	SAM@O3′.HO3′	2.66	46.27
SAM@HN1	Tyr47@OH.HH	2.78	45.41
Asp99@OD1	SAM@O2′.HO2′	2.63	45.15
SAM@O	Asn43@ND2.HD22	2.84	42.20
SAM@OXT	Asn43@ND2.HD22	2.84	37.76
SAM@N1	Cys115@N.H	2.92	33.86
Asp114@OD2	SAM@N6.HN61	2.80	18.10
Asp114@OD1	SAM@N6.HN61	2.80	15.11
SAM@O2′	Asn101@ND2.HD22	2.89	12.21
**nsp16**_**SAM**_			
Asp99@OD1	SAM@O3′.HO3′	2.64	48.80
Asp99@OD2	SAM@O3′.HO3′	2.63	45.78
SAM@N1	Cys115@N.H	2.92	28.66
Asp114@OD2	SAM@N6.HN61	2.80	26.44
Asp114@OD1	SAM@N6.HN61	2.80	24.80
Asp114@OD2	SAM@N6.HN62	2.80	19.03
Asp114@OD1	SAM@N6.HN62	2.80	17.95
Asp99@OD1	SAM@O2′.HO2′	2.65	16.42
Asp99@OD2	SAM@O2′.HO2′	2.65	14.70
SAM@N	Tyr47@OH.HH	2.92	11.31

a *only H-bonds with more than 10% occupancy are listed*.

*The corresponding average distance and percentage occupancy are also provided*.

The changes in the distance of the atoms forming H-bond between SAM and nsp16 were also determined and shown in Supplementary Figure 6. In the complex, the distance between the oxygen atom of Asp130, Tyr47, and Gly71 of nsp16 and the nitrogen atom of SAM illustrated the average distance of 3.01, 2.89, and 3.01 Å, respectively, suggesting strong H-bond interactions. In the case of nsp16_SAM_, the average distances for these atoms were 12.48, 14.64, and 11.06 Å, respectively, indicating a lack of formation of H-bonds in the nsp16 monomer. However, for Asp99, the distance between its oxygen atom (OD1 and OD2) and the oxygen atom (O3) of SAM illustrated an average distance of 2.9 Å and 3.2 Å for the complex and monomer, respectively. In contrast, the average distance between Asp99 (OD2) and SAM (O2) was higher for monomer (4.2 Å) than the complex (3.1Å). All these results were consistent with the occupancy analysis. Further, they emphasized that Asp130, Tyr47, and Gly71 of nsp16 were key residues in the binding of SAM and for the 2′-O-MTase activity of nsp16.

### Energetics of nsp16/nsp10 Complexation

To evaluate further the binding free energy of the heterodimer (nsp16/nsp10), Δ*G*_bind_ was estimated using the MD/MM-PBSA approach and reported in Table 5. From Table 5, the binding free energy (Δ*G*_bind_) between nsp16 and nsp10 was found to be −47.4 kcal/mol. The various components of the total binding free energy indicate that the intermolecular van der Waals interactions (Δ*E*_vdW_), electrostatic interactions (Δ*E*_ele_), and non-polar solvation free energy (Δ*G*_np_) favors the binding of nsp10 and nsp16. While polar solvation free energy (Δ*G*_pol_) disfavor the binding. The electrostatic interactions (Δ*E*_ele_) energy is higher (−429.4 kcal/mol) than the van der Waal interactions (Δ*E*_vdW_) (−90.4 kcal/mol) (see Supplementary Figure 7). However, the disfavouring components of polar solvation energy (Δ*G*_pol_) compensate for the Δ*E*_ele_ being a value of 481.7 kcal/mol. Hence, the total polar interactions (Δ*E*_ele_ + Δ*G*_pol_) disfavor the binding between nsp10 and nsp16 with a value of 52.3 kcal/mol. In contrast, the total non-polar contributions (Δ*E*_vdW_ + Δ*G*_np_) favor the binding between nsp16 and nsp10 (−100.0 kcal/mol). Therefore, the binding between nsp10 and nsp16 is mainly driven by hydrophobic interactions. Overall, the binding free energy analysis depicts, the binding affinity of the SAM in the complex nsp16/nsp10 (Δ*G*_bind_ = −46.6 kcal/mol) is similar to that between nsp10 and nsp16 (Δ*G*_bind_ = −47.4 kcal/mol) of SARS-CoV-2. These observations were in agreement with its similar homology virus, SARS-CoV (2002). A similar binding affinity was seen between nsp10 and nsp16, as well as between SAM and the nsp16/nsp10 complex of SARS-CoV (Chen et al., 2011).

**Table 5:** Energetic components of the dimerization energy (*G*_*bind*_) between nsp16 and nsp10.

**System**	**ΔE_vdW_**	**ΔE_elec_**	**ΔG_pol_**	**ΔG_np_**	**ΔG_bind_**	**$\Delta {\text{G}}_{\text{bind}}^{\text{a}}$**
WT	−90.4 (0.2)	−429.4 (3.0)	481.7 (3.2)	−9.6 (0.0)	−47.7 (0.4)	
I40A	−86.0 (0.2)	−429.2 (3.0)	480.5 (3.2)	−9.5 (0.0)	−44.2 (0.4)	3.5 (1.2)
V44A	−88.0 (0.2)	−429.2 (3.0)	481.4 (3.2)	−9.6 (0.0)	−45.5 (0.4)	2.1 (1.5)
V78A	−87.5 (0.2)	−429.5 (3.0)	481.0 (3.2)	−9.5 (0.0)	−45.5 (0.4)	2.2 (1.5)
R86A	−87.5 (0.2)	−522.6 (3.0)	574.5 (3.3)	−9.5 (0.0)	−45.1 (0.4)	2.6 (2.5)
Q87A	−86.8 (0.2)	−416.9 (3.0)	466.8 (3.2)	−9.4 (0.0)	−46.2 (0.4)	1.5 (1.6)
V104A	−86.9 (0.2)	−429.7 (3.0)	481.11 (3.2)	−9.4 (0.0)	−44.9 (0.4)	2.8 (1.5)
M247A	−87.1 (0.2)	−428.5 (3.0)	479.0 (3.2)	−9.6 (0.0)	46.1 (0.4)	1.5 (0.8)

*Along with the Computational Alanine-Scanning (CAS) mutagenesis results of the complex nsp16/nsp10. The binding energy is given in kcal/mol, and the standard error of the mean is provided in parentheses*.

$\Delta \Delta G_{bind}^{a}$ = $\Delta G_{bind}^{mut}-\Delta G_{bind}^{WT}$.

**Figure 7:**
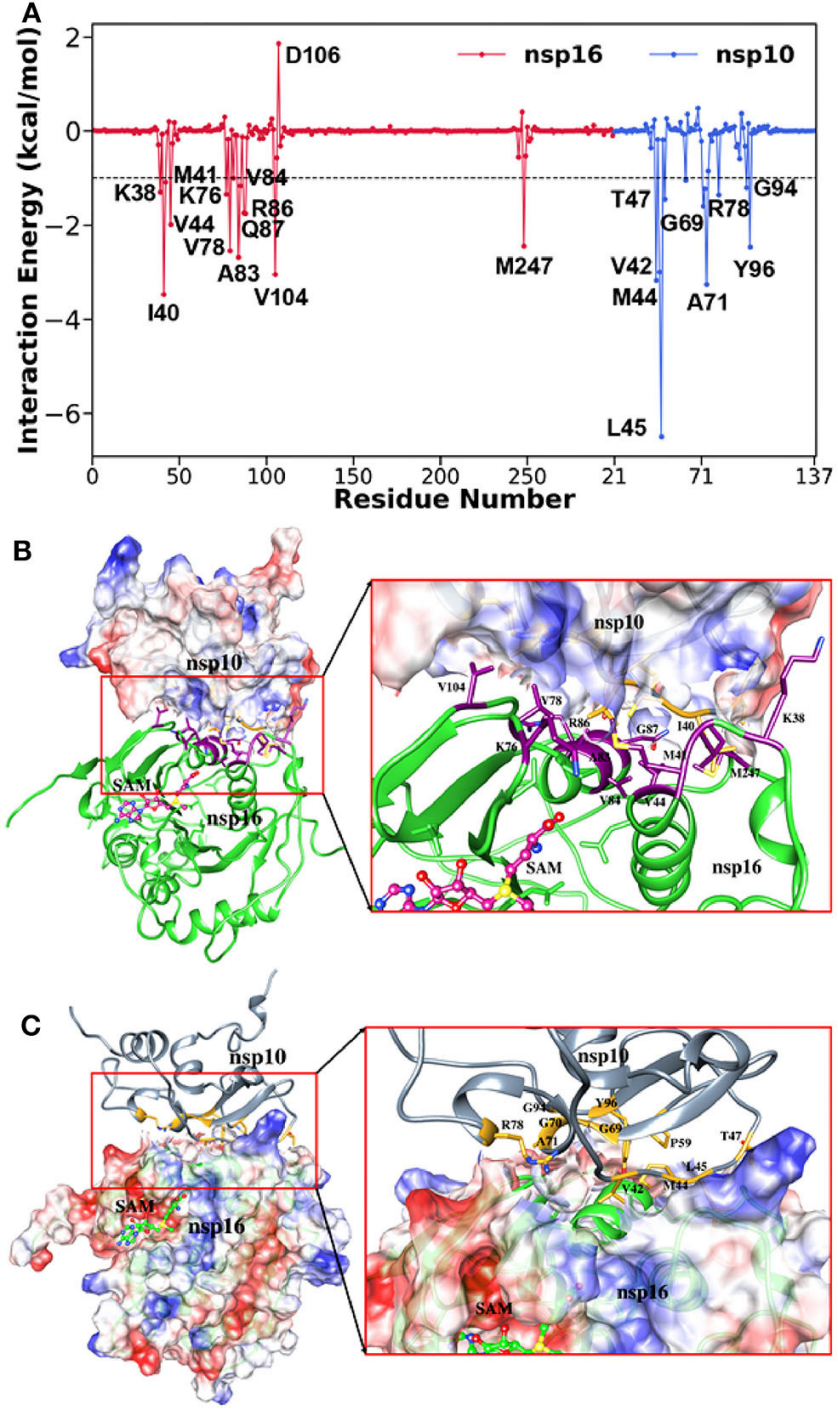
**(A)** Decomposition of Δ*G* on a per-residue basis for the complex, **(B)** corresponding residual position of nsp16 in complex, and **(C)** residual position of nsp10 in the complex.

To further investigate the critical residues involved in the binding between nsp10 and nsp16, the binding free energy was decomposed at the individual residue level using the MM-GBSA approach and shown in Figure 7. Here, we considered only residues having the energy value ≥ 1.0 kcal/mol and listed in Supplementary Table 3. The key residues from nsp10 include Leu45, Ala71, Val42, Met44, Tyr96, Gly69, Thr47, Arg78, Gly70, Gly94, and Pro59. Similarly, the critical residues from nsp10 include Ile40, Val104, Ala83, Val78, Met247, Val44, Gln87, Arg86, Lys76, Lys38, Val84, and Met41. It indicates that hydrophobic interactions significantly control the binding between nsp10 and nsp16. Our findings agree with a recent experimental study (Lin S. et al., 2020) where it was reported that a total of 31 residues (Asn40, Val42, Lys43, Met44, Leu45, Cys46, Thr47, Pro59, Gly70, Ala71, Cys77, Arg78, Lys93, Gly94, and Tyr96 in nsp10 and Lys38, Gly39, Ile40, Met41, Val44, Lys76, Val78, Pro80, Ala83, Arg86, Gln87, Val104, Ser105, Asp106, Leu244, and Met247 in nsp16) mediates the intimate nsp16-nsp10 interaction. The per-residue decomposition of the binding free energy was further validated by other computational approaches, such as FTmap (Kozakov et al., 2015). The final complex structure obtained from the MD trajectory was analyzed using FTmap, and Gln87, Arg86, Val104, Met247 of nsp16, and Leu45, Met44, Pro59, Arg78, Tyr96 of nsp10 were identified as the hotspot residues, which again agrees with the MM-GBSA analysis.

### Interaction Analysis Between nsp16 and nsp10

**Figure 8:**
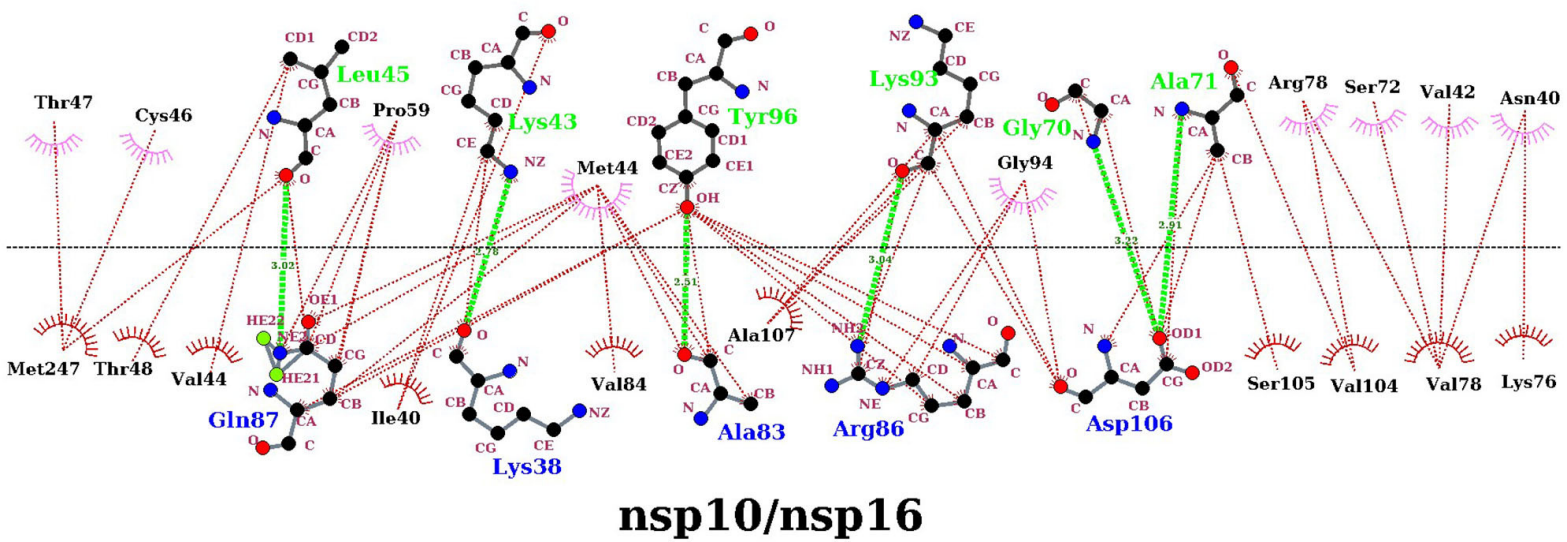
Protein-protein interaction diagrams for the nsp16/nsp10 dimerization. The upper panel corresponds to nsp10, and the lower panel corresponds to nsp16 in the complex. The plot was generated with the dimplot module of LigPlot+. Hydrogen bonds are shown as a lime green dotted line, and hydrophobic bonds are represented in red.

In the above finding of binding free energy analysis, we have seen that nsp10 acts as a stimulator in the binding of the SAM to nsp16. Hence, to further explore the binding interactions between nsp16 and nsp10, H-bond and hydrophobicity analysis was calculated. As seen in Table 6, the H-bond occupancy reflects the stability of H-bond formation between protein-protein in the MD simulations. The strong H-bonds were formed between residues Ala83 (nsp16) and Tyr96 (nsp10) and between Gln87 (nsp16) and Leu45 (nsp10). Other residues, including Asp106 (nsp16), formed two H-bonds with Ala71 and Gly94 of nsp10, Lys38 (nsp16) to Lys43 (nsp10), and Ser105 (nsp16) to Lys93 (nsp10). Hydrophobic interactions also played an important role in protein-protein or protein-ligand interactions (Jonniya et al., 2020; Roy et al., 2020; Sk et al., 2020d). Different H-bonds and hydrophobic interactions from the stable structure of the nsp16-nsp10 obtained from the MD simulations were plotted via Ligplot (Wallace et al., 1995) and shown in Figure 8.

**Table 6:** The hydrogen bonds formed between nsp16 and nsp10^*^ in the complex and the corresponding average distance and percent determined using the production trajectories in the MD simulations.

**Binding couples**		**Molecular dynamics**	
**Acceptor**	**Donor…H**	**Distance (Å)**	**Occupancy (%)**
Ala83@O	**Tyr96@OH…HH**	2.73	90.80
**Leu45@O**	Gln87@NE2…HE21	2.85	67.83
Asp106@OD1	**Ala71@N…H**	2.87	29.23
Asp106@OD1	**Gly94@N…H**	2.84	24.23
Lys38@O	**Lys43@NZ…HZ2**	2.80	25.28
Lys38@O	**Lys43@NZ…HZ3**	2.80	25.01
Lys38@O	**Lys43@NZ…HZ1**	2.80	23.63
Asp106@OD2	**Gly94@N…H**	2.84	17.58
Ser105@O	**Lys93@NZ…HZ3**	2.79	12.65
Ser105@O	**Lys93@NZ…HZ2**	2.79	12.35
Ser105@O	**Lys93@NZ…HZ1**	2.79	12.25

* *Bold letters belong to the nsp10 structure. Only H-bonds with more than 10% occupancy are listed*.

The percentage occupancy of the residual contacts with the cut-off 3.9 Å for the protein-protein is also listed in Supplementary Table 4. Overall, these results highlighted the significant residues forming strong interactions between nsp16 and nsp10, which may aid in the development and design of inhibitors that could block these protein-protein interactions by inhibiting the 2′-O-MTase activity.

### Computational Alanine Scanning

**Figure 9:**
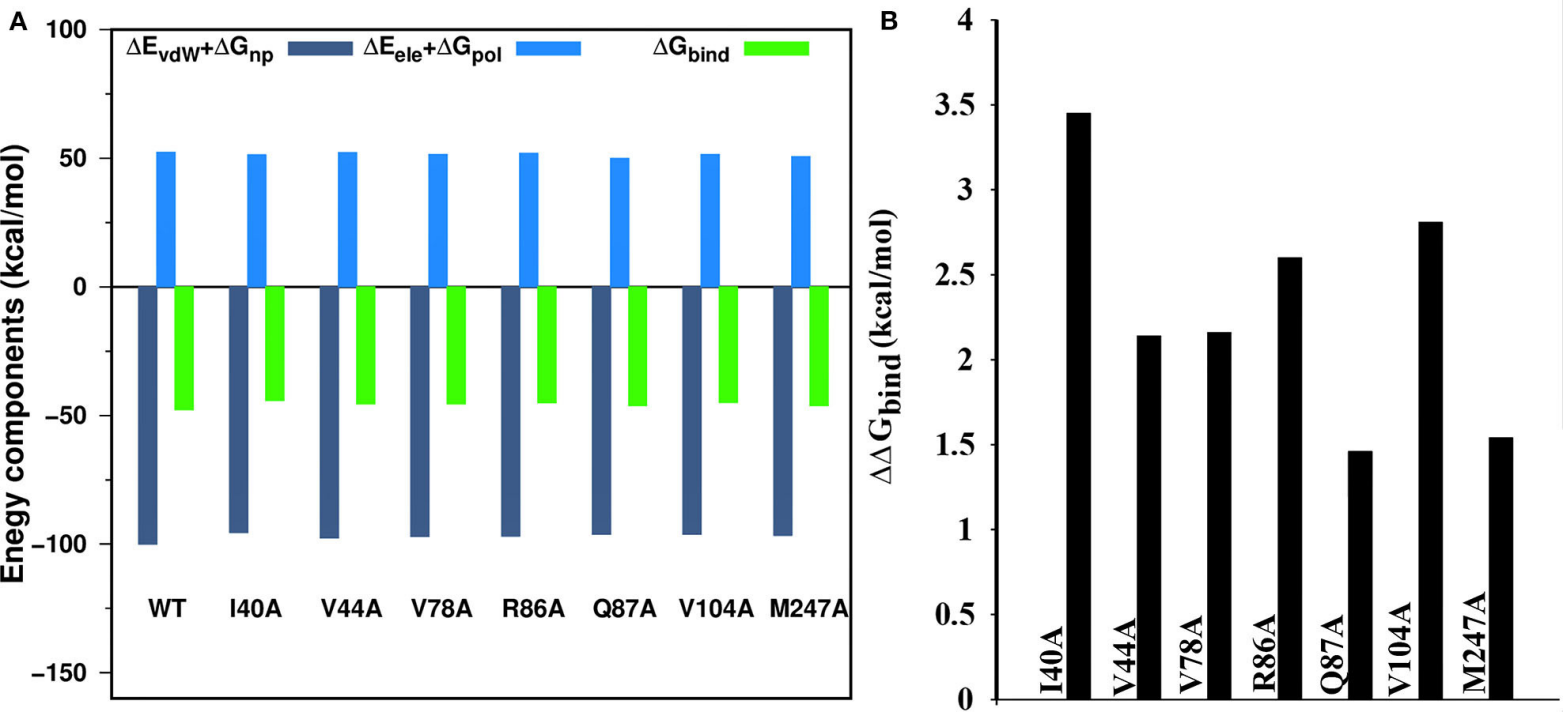
**(A)** Binding free energy components of the wild type and seven mutations (alanine scanning) in the complex, **(B)** alanine scanning mutagenesis analysis of complex.

The changes in the binding free energy were computed as in Equation (4) (see Method section) after replacing the residue of WT to alanine. The impact of a single mutation in the protein-protein interaction is mainly reflected by the more positive value of the ΔΔ*G*_*bind*_. In our study, we have conducted CAS for nsp16 by considering those residues with the decomposition free energy > 1.5 kcal/mol, as given in Supplementary Table 3. The binding free energy components calculated from the CAS mutagenesis for residues I40A, V104A, R86A, V78A, V44A, M247A, and Q87A of nsp16 are listed in Table 5 and compared with the WT. As seen in Supplementary Table 3, although the Δ*G*_bind_ of WT is higher than mutants, different energy components of mutants follow the same trend as WT. The Δ*E*_ele_ is higher than Δ*E*_vdW_, but disfavouring polar solvation energy Δ*G*_pol_ compensates Δ*E*_ele_. As shown in Figure 9A, for all the systems, the binding free energy is mainly coming from the hydrophobic interactions, where the total non-polar energy (Δ*E*_vdW_ + Δ*G*_np_) is higher than the total polar energy (Δ*E*_ele_ + Δ*G*_pol_). Figure 9B reflects significant residues in the protein interface calculated from CAS mutagenesis. It shows that ΔΔ*G*_*bind*_ for mutants, I40A, V104A, and R86A are comparatively high, suggesting that primarily these residues play a significant role in the heterodimer formation, which is in accordance with the decomposition of energy. The CAS mutagenesis results depict that in addition to hydrophobic residues I40 and V104, the hydrophilic residue R86 also plays a vital role in the binding of nsp10-nsp16.

### Temperature and Salt Concentration Effect on the Binding Affinity

Next, we investigated the effect of temperature and salt concentration in the binding of nsp16/nsp10 and nsp16/nsp10/SAM using the MM-PBSA method. This method presents itself to be a perfect model to study the effect of conformational dynamics of the same protein evolved in different environmental conditions to the fate of the ligand, ultimately decided by the binding affinity or dissociation.

Supplementary Tables 5, 6 displayed the binding free energy components for nsp16-nsp10 and nsp16/nsp10/SAM, respectively, at two different temperatures. As shown in Supplementary Table 5, the predicted binding free energies between nsp10 and nsp16 (Δ*G*_bind_) was found to be −47.7 kcal/mol, and −52.2 kcal/mol at 300 and 310 K, respectively. It suggests that nsp16 binds more strongly to nsp10 at 310 K than 300 K. It can also be noted from Supplementary Table 5 that at the elevated temperature (310 K), the magnitude of total polar interactions (Δ*E*_ele_ + Δ*G*_pol_) decreases (52.3 to 49.6 kcal/mol) and oppose less unfavorably to the protein-protein association. At a higher temperature, the non-polar interactions (Δ*E*_vdW_ + Δ*G*_np_) decreased from −100 to −102 kcal/mol and contributed more favorably to the association of nsp16-nsp10. Similarly, Supplementary Table 6 suggested that the estimated binding free energies (without entropy) of the cofactor SAM with nsp16/nsp10 (Δ*G*_bind_) were −46.6 and −13.2 kcal/mol at 300 and 310 K, respectively. It was further evident from Supplementary Table 6 that after increasing the temperature, the binding affinity of SAM toward nsp16/nsp10 decreased. At room temperature (300 K) both total polar (Δ*E*_ele_ + Δ*G*_pol_ = −2.1 kcal/mol) and non-polar (Δ*E*_vdW_ + Δ*G*_np_ = −44.5 kcal/mol) interactions favored the binding of SAM with nsp16. After increasing the temperature to 310 K, the total polar interaction (Δ*E*_ele_ + Δ*G*_pol_) became unfavorable to the binding of SAM to nsp16/nsp10. However, the total non-polar contribution (Δ*E*_vdW_ + Δ*G*_np_) still favored the binding. As compared to room temperature, the strength of the non-polar (Δ*E*_vdW_ + Δ*G*_np_) interactions decreases significantly (−44.5 to −23.5 kcal/mol). At 310 K, we have noticed that up to 400 ns, SAM binds strongly to nsp16. After that, SAM got detached from the binding cavity (see Supplementary Figure 8).

The salt concentration also influenced the protein-protein interactions as well as the binding of SAM to nsp16/nsp10, which are evident from Supplementary Tables 5, 6. As shown in Supplementary Table 5, the predicted binding free energies of nsp10 with nsp16 (Δ*G*_bind_) were found to be −44.8 and −37.7 kcal/mol, for the salt concentration of 0.15 and 0.25 M, respectively. After increasing the salt concentration, it was observed that the total polar (Δ*E*_ele_ + Δ*G*_pol_) and non-polar interactions (Δ*E*_vdW_ + Δ*G*_np_) increased from 54.1 to 55.3 kcal/mol and −98.9 to −93 kcal/mol, respectively. The rise in unfavorable interactions caused the destabilization of the protein-protein interactions. Similarly, the binding of SAM to the heterodimer was found to be affected by the increased salt concentration (see Supplementary Table 6). The estimated binding free energy of SAM (Δ*G*_bind_) was −23.7 and −22.1 kcal/mol for the salt concentration of 0.15 and 0.25 M, respectively. These values are significantly higher than what was obtained for the neutral salt concentration (−46.6 kcal/mol). Δ*E*_vdW_ and Δ*E*_elec_ were found to contribute less favorably to the binding of SAM to nsp16/nsp10 in comparison to neutral simulations. A similar trend was observed when the temperature was increased from 300 to 310 K. The time evolution of CoM distance between nsp16 and SAM was displayed in Supplementary Figure 9. It is evident from Supplementary Figure 9 that with the increased salt concentration, the CoM distance between nsp16 and SAM increased and remained stable around 17 Å and 18 Å for 0.15 and 0.25 M, respectively. This implies that the cofactor SAM moves away from the binding cavity with the increased salt concentration.

## Conclusions

Herein, we have employed extensive MD simulations of 1 μs along with the molecular mechanics/Poisson-Boltzmann surface area (MM/PBSA) method to study the binding mechanism of the cofactor SAM to the nsp16/nsp10 heterodimer and sub-unit nsp16 alone (nsp16_SAM_) of SARS-CoV-2. Our MD/MMPBSA calculations suggested that SAM binds strongly to the nsp16/nsp10 heterodimer and fails to bind to the nsp16 monomer. It emphasized that nsp10 helps in the strong interaction between SAM and nsp16 to execute the 2′-O-MTase activity. This finding agrees well with the experimental study, and a similar observation was made for other coronaviruses, including SARS-CoV and MERS-CoV. We have also investigated the interactions between nsp16 and nsp10. Our study revealed that the binding of nsp10 stabilizes the complex (nsp16/nsp10) structure. Further, our study showed that hydrophobic interactions are critical for the heterodimer association. Thus, apart from the active site of nsp16, the interface of nsp16/nsp10 can also be considered as a potential target site in the design of antiviral drugs such as peptide inhibitors. It includes Ile40, Val104, Ala83, Val78, Met247, Val44, Gln87, Arg86, Lys76, Lys38, Val84, and Met41 from nsp16, and Leu45, Ala71, Val42, Met44, Tyr96, Gly69, Thr47, Arg78, Gly70, Gly94, and Pro59 from nsp10. Besides, the stable hydrogen bond between Ala83 (nsp16) and Tyr96 (nsp10), and between Gln87 (nsp16) and Leu45 (nsp10) were important in the nsp16-nsp10 interface. The CAS study reveals that residues I40A, V104A, R86A, V78A, V44A, M247A, and Q87A of nsp16 were considered as hot spot residues for the association of nsp16-nsp10. ΔΔ*G*_*bind*_ for mutants, I40A, V104A, and R86A are comparatively high, suggesting the significance of these residues. Finally, we investigated the effect of temperature and salt concentration on the binding of SAM to the heterodimer. Our study revealed that the SAM binding was impaired due to an increase in temperature or salt concentration. Finally, our study provides a comprehensive understanding of the dynamic and thermodynamic process of binding nsp16 and nsp10 that will contribute to the rational design of inhibitors targeting nsp16/nsp10.

## Data Availability Statement

The raw data supporting the conclusions of this article will be made available by the authors, without undue reservation.

## Author Contributions

Project conceived and supervised by PK. Simulations and analyses were performed by MS, NJ, RR, and SP. Manuscript was written by MS, NJ, and PK. All authors contributed to the article and approved the submitted version.

## Conflict of Interest

The authors declare that the research was conducted in the absence of any commercial or financial relationships that could be construed as a potential conflict of interest.
