# Supplementary material for: Computational Investigation of Structural Dynamics of SARS-CoV-2 Methyltransferase-Stimulatory Factor Heterodimer nsp16/nsp10 Bound to the Cofactor SAM
Source: Front Mol Biosci. 2020 Nov 24;7:590165. doi: 10.3389/fmolb.2020.590165 (PMC7732651; doi:10.3389/fmolb.2020.590165)
Supplement: Supplementary file 1 [file Data_Sheet_1.PDF]

Supplementary Information

Computational investigation of structural dynamics of SARS-CoV-2 methyltransferase-stimulatory factor heterodimer nsp16/nsp10 bound to the cofactor SAM

Md Fulbabu Sk[#], Nisha Amarnath Jonniya[#], Rajarshi Roy, Sayan Poddar, Parimal Kar*

Discipline of Biosciences and Biomedical Engineering, Indian Institute of Technology Indore, Khandwa Road, MP 453552, India

[#]Both authors contributed equally

*Corresponding author: Parimal Kar| Email: parimal@iiti.ac.in| Phone: +91 731 2438700 (Ext. 550)

Table S1: The average RMSD values of various regions and SAM for the complex and $nsp16_{SAM}$ systems. All values are in Å unit, and standard deviations are given in parentheses.

Region/cofactor	complex	$nsp16_{SAM}$
SAM	0.7 (0.1)	2.1 (0.8)
Around 5Å of SAM	0.8 (0.1)	2.2 (0.8)
Loop 26-38 (last 400 ns)	1.4 (0.5)	3.1 (0.5)

Table S2: The hydrophobic network at the interface of nsp10 and nsp16 in the complex.

Structure	Hydrophobic network nodes
0 ns	(V104, **A71**), (V78, **V42**), (P80, **V42**), (A83, **V42**), (A83, **M44**), (I40, **L45**), (L244, **L45**), (M247, **L45**)
100 ns	(V104, **A71**), (P80, **V42**), (M247, **L45**)
200 ns	(I40, **M44**), (V104, **A71**)
300 ns	(V104, **A71**), (P80, **V42**)
400 ns	(V104, **A71**), (P80, **V42**)
500 ns	(V104, **A71**), (P80, **V42**), (I40, **M44**)

Bold letters residues belong to nsp10

Table S3: Binding free energy decomposition of key residues between nsp16 and nsp10 in complex in the unit of kcal/mol.

Residue	T_{vdW}	T_{ele}	T_{pol}	T_{np}	T_{back}	T_{side}	T_{total}
nsp16							
Ile40	-3.21	0.07	0.11	-0.44	-0.22	-3.25	-3.47
Val104	-3.40	3.00	-1.98	-0.67	0.29	-3.34	-3.05
Ala83	-1.47	-1.84	0.92	-0.29	-1.31	-1.37	-2.68
Val78	-2.66	1.61	-1.05	-0.44	0.09	-2.63	-2.54
Met247	-3.20	-1.12	2.33	-0.46	0.16	-2.61	-2.45
Val44	-1.87	-0.03	0.18	-0.27	-0.05	-1.94	-1.99
Gln87	-3.22	-4.41	6.54	-0.66	0.37	-2.12	-1.75
Arg86	-2.37	48.08	-47.12	-0.33	-0.60	-1.14	-1.74
Lys76	-1.58	27.69	-27.02	-0.43	-0.34	-1.00	-1.34
Lys38	-0.43	3.01	-3.60	-0.27	-0.93	-0.36	-1.29
Val84	-1.11	0.44	-0.46	-0.03	-0.44	-0.72	-1.16
Met41	-1.25	-0.13	0.50	-0.20	-0.02	-1.06	-1.08
nsp10							
Leu45	-5.75	-2.51	2.98	-1.22	-0.83	-5.67	-6.50
Ala71	-2.49	-5.31	4.97	-0.42	-1.21	-2.04	-3.25
Val42	-3.23	1.06	-0.38	-0.62	0.09	-3.26	-3.17
Met44	-3.86	-1.24	2.50	-0.39	0.16	-3.15	-2.99
Tyr96	-2.39	-3.63	4.00	-0.44	0.04	-2.50	-2.46
Gly69	-0.73	-3.60	2.81	-0.07	-1.36	-0.23	-1.59
Thr47	-1.87	0.06	0.77	-0.41	-0.29	-1.16	-1.45
Arg78	-2.38	-31.70	33.16	-0.44	0.14	-1.49	-1.36
Gly70	-0.64	-3.58	3.02	-0.02	-1.11	-0.11	-1.22
Gly94	-1.76	-3.85	4.75	-0.34	-0.59	-0.61	-1.20
Pro59	-1.28	-1.46	1.88	-0.17	-0.18	-0.85	-1.03

Table S4: Percentage atomic contacts between nsp16 and nsp10 in a complex from the MD simulations and set the cutoff distance is 3.90 Å.

Contacts		Molecular Dynamics (MD)	
nsp16	nsp10	Occupancy (%)	Avg. Distance (Å)
Ala83@O	Tyr96@OH	99.6	2.8
Gln87@N	Tyr96@OH	99.2	3.1
Gln87@NE2	Leu45@O	99.2	2.9
Arg86@CB	Tyr96@OH	98.1	3.4
Arg86@C	Tyr96@OH	98.0	3.3
Lys38@O	Lys43@NZ	97.8	2.8
Gln87@CA	Tyr96@OH	92.4	3.5
Ser105@C	Ala71@CB	92.4	3.4
Lys38@O	Lys43@CE	91.2	3.6
Arg86@CG	Tyr96@OH	90.9	3.5
Gln87@CB	Tyr96@OH	86.5	3.6
Ser105@O	Ala71@CB	82.9	3.5
Ala81@O	Met44@CE	82.7	3.5
Gln93@OE1	Met44@CE	82.6	3.4
Ala83@C	Met44@CE	75.2	3.7
Gln87@NE2	Pro59@CG	75.1	3.6
Asp106@OD1	Ala71@CB	71.5	3.5
Asp106@OD1	Ala71@N	70.2	3.1
Gln87@CD	Leu45@O	64.0	3.7
Asp106@OD1	Gly70@N	62.9	3.4
Asp106@O	Gly94@CA	59.9	3.5
Gln87@CD	Pro59@CG	59.4	3.7
Asp106@CG	Ala71@CB	59.1	3.7
Arg86@CA	Tyr96@OH	56.9	3.8
Thr48@OG1	Leu45@CD1	56.3	3.6
Gln87@OE1	Leu45@O	53.2	3.7
Asp106@OD2	Ala71@CB	50.6	3.6

Only contacts with more than 50% occupancy are listed.

Table S5: Energetics components of binding free energy between nsp16 and nsp10 in the presence of cofactor SAM in a complex.

Complex at	ΔE_{vdW}	ΔE_{elec}	ΔG_{pol}	ΔG_{np}	ΔG_{bind}
300 K	-90.4 (0.2)	-429.4 (3.0)	481.7 (3.2)	-9.6 (0.0)	-47.7 (0.4)
310 K	-91.9 (0.2)	-279.3 (1.5)	328.9 (1.5)	-9.9 (0.0)	-52.2 (0.3)
0.15 M, 300 K	-89.6 (0.1)	-247.5 (1.2)	301.6 (1.2)	-9.3 (0.0)	-44.8 (0.2)
0.25 M, 300 K	-83.8 (0.2)	-212.3 (1.3)	267.6 (1.4)	-9.2 (0.0)	-37.7 (0.2)

Table S6: Energy components of the binding free energy between the cofactor SAM and nsp16 in the presence of nsp10 in a complex.

Complex at	ΔE_{vdW}	ΔE_{elec}	ΔG_{pol}	ΔG_{np}	ΔG_{bind}
300 K	-40.4 (0.1)	-181.1 (0.5)	179.0 (0.4)	-4.1 (0.0)	-46.6 (0.2)
310 K	-21.0 (0.1)	-44.1 (0.8)	54.5 (07)	-2.5 (0.0)	-13.2 (0.2)
0.15 M, 300 K	-29.0 (0.1)	-69.9 (0.5)	78.7 (0.4)	-3.5 (0.0)	-23.7 (0.1)
0.25 M, 300 K	-29.0 (0.1)	-68.5 (0.5)	78.9 (0.5)	-3.5 (0.0)	-22.1 (0.1)

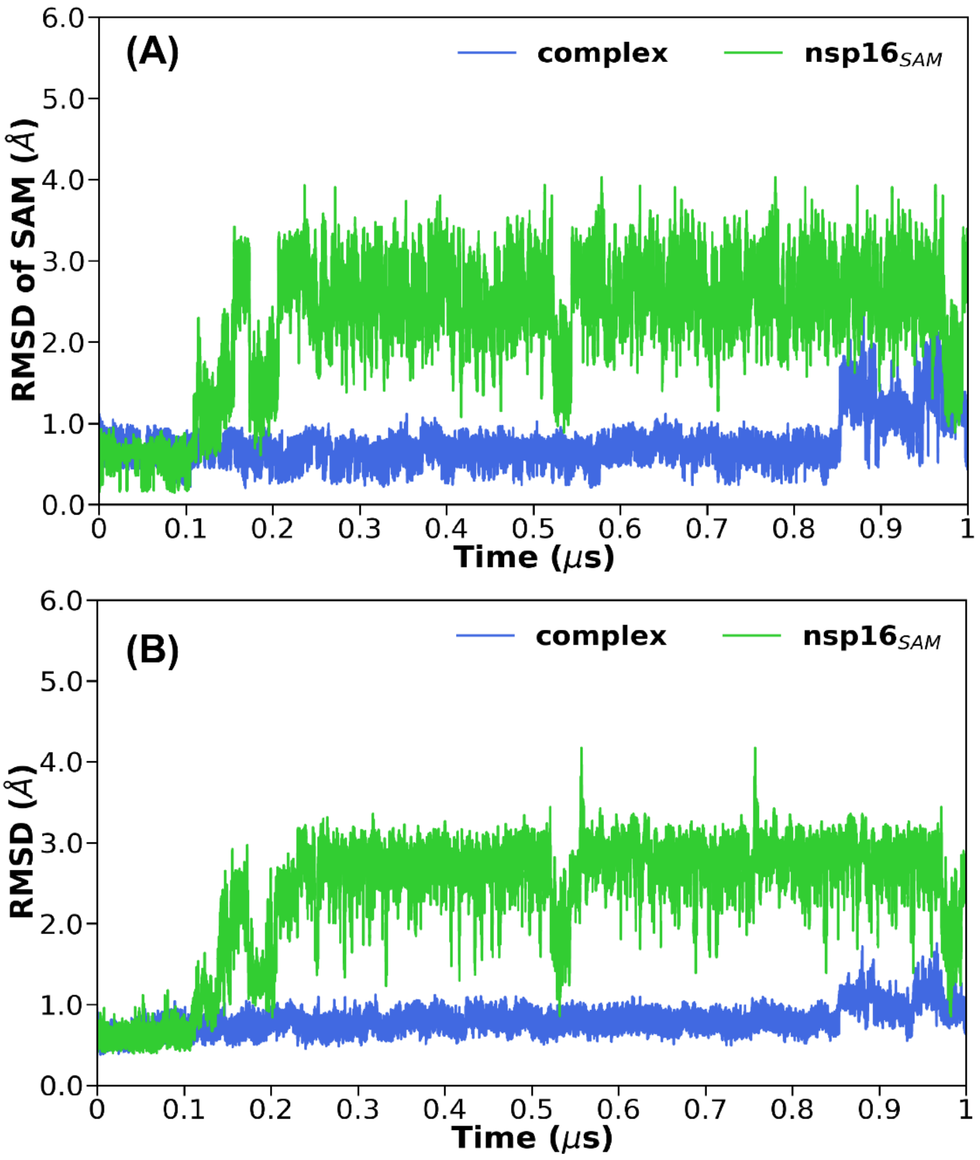

Figure S1: The time evolution of root-mean-square deviations (RMSDs) of (**A**) heavy atoms of SAM and (**B**) binding pocket (5Å radius of SAM) of the complex and $nsp16_{SAM}$ systems.

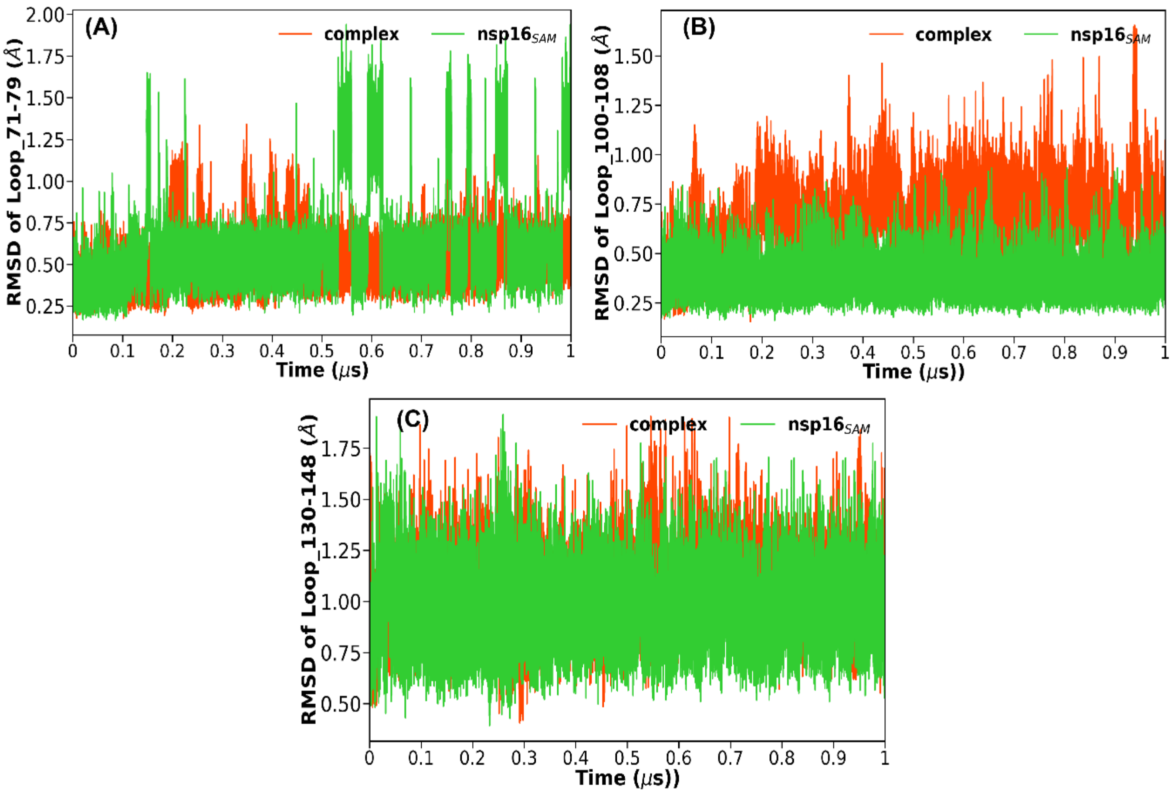

Figure S2: The temporal RMSDs of backbone atoms of **(A)** loop 71-79, **(B)** loop 100-108, and **(C)** loop 130-148 in the complex (orange-red) and nsp16$_{SAM}$ (lime-green) form.

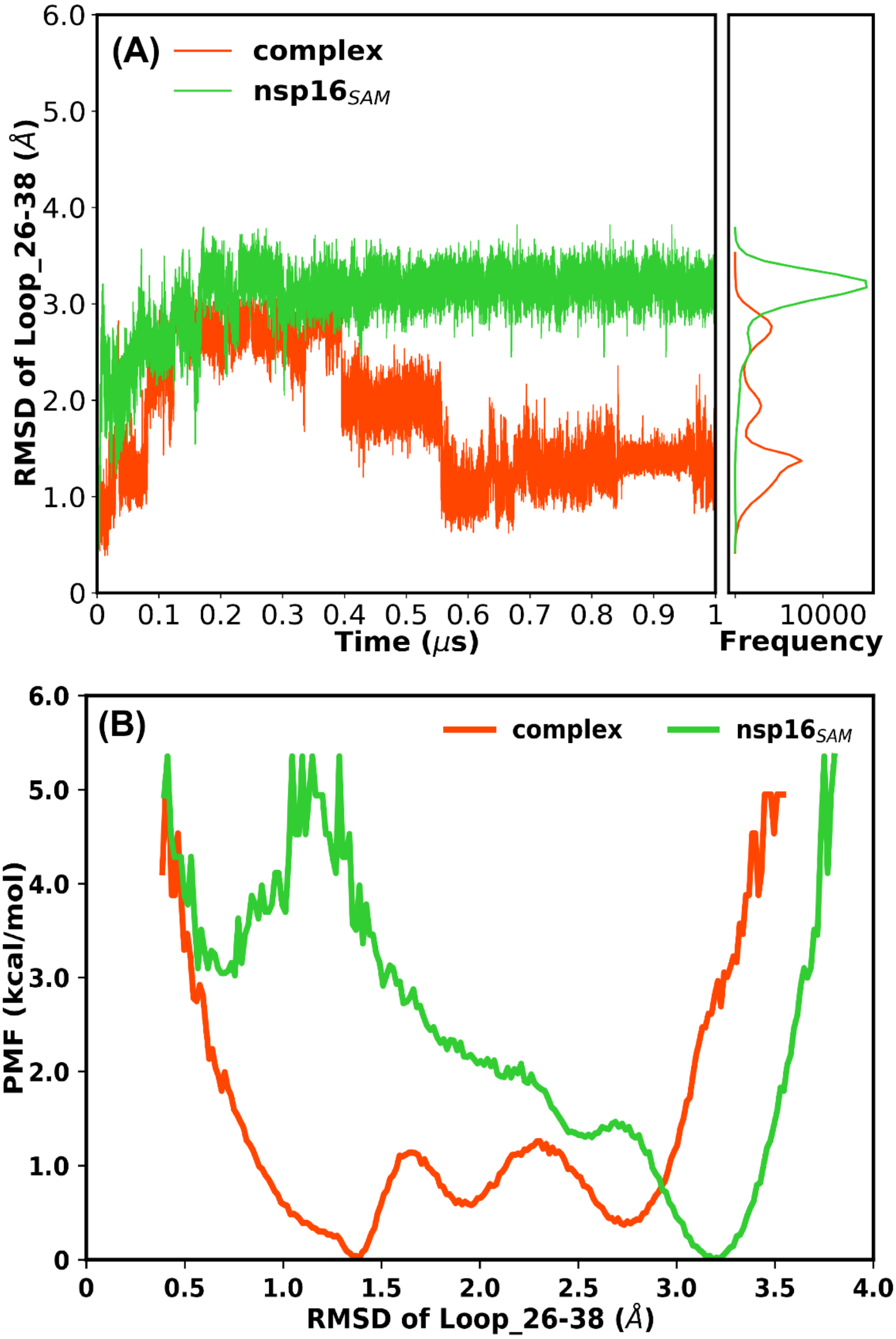

Figure S3: (**A**) The temporal RMSDs of backbone atoms of loop 26-38 in the complex (orange-red) and nsp16$_{SAM}$ (lime-green) form, (**B**) the potential of mean force (PMF) of loop 26-38 by calculated the free energy profile along with the backbone atoms RMSD values of loop 26-38.

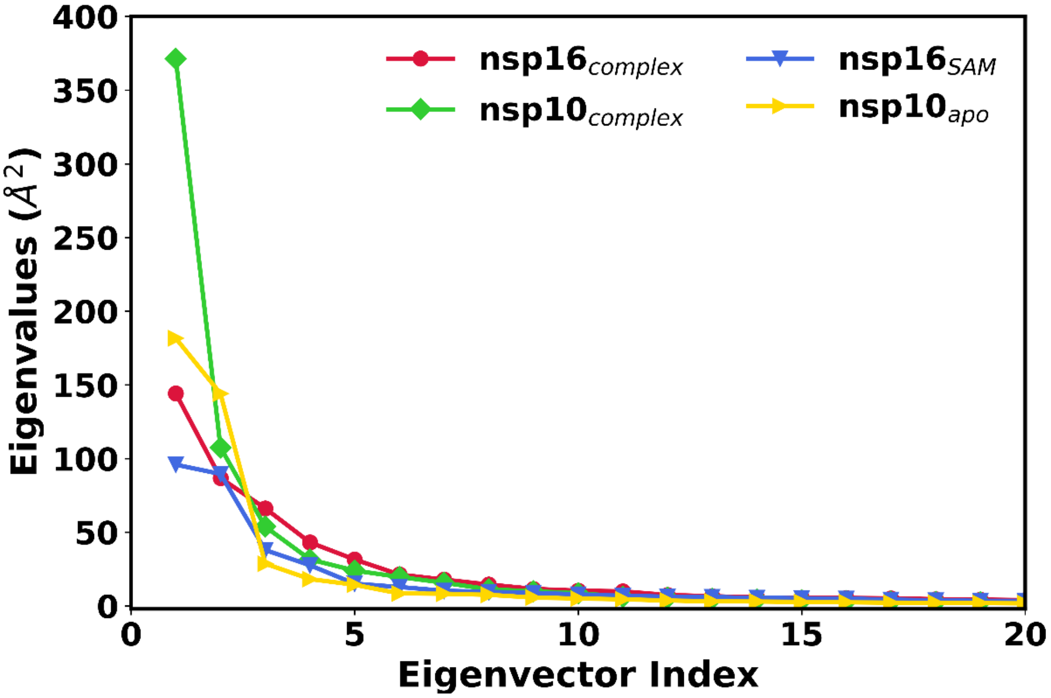

Figure S4: Comparison of the eigenvalues plotted against the corresponding eigenvector indices for nsp16$_{complex}$ (crimson), nsp10$_{complex}$ (lime-green), nsp16$_{SAM}$ (sky-blue) and nsp10$_{apo}$ (gold).

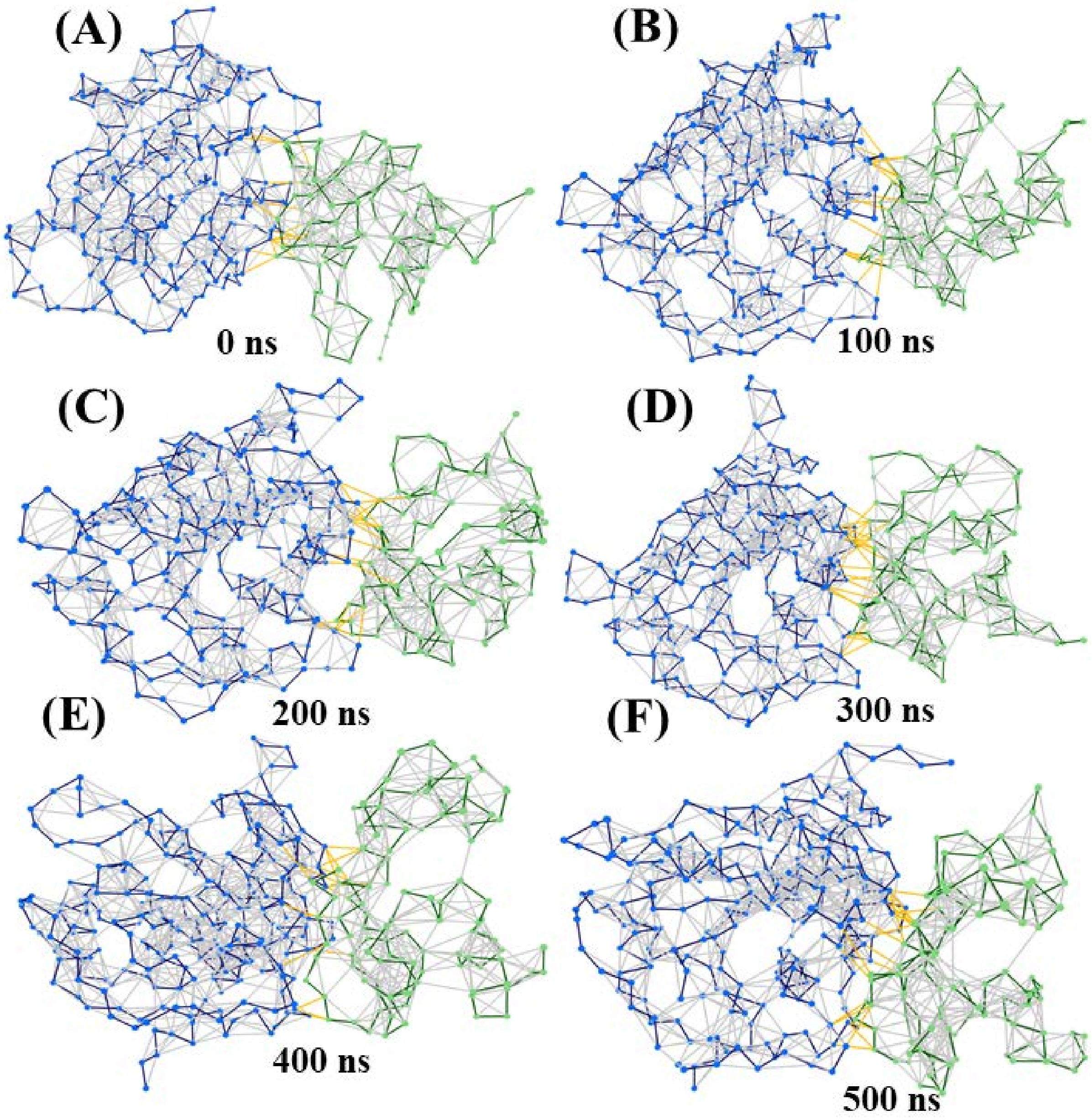

Figure S5: The residue contact network analysis between nsp16 and nsp10 in a complex during the simulation. The total number of interaction networks are conserved for each case. The yellow line edges represent contact between nsp16 (blue) and nsp10 (green).

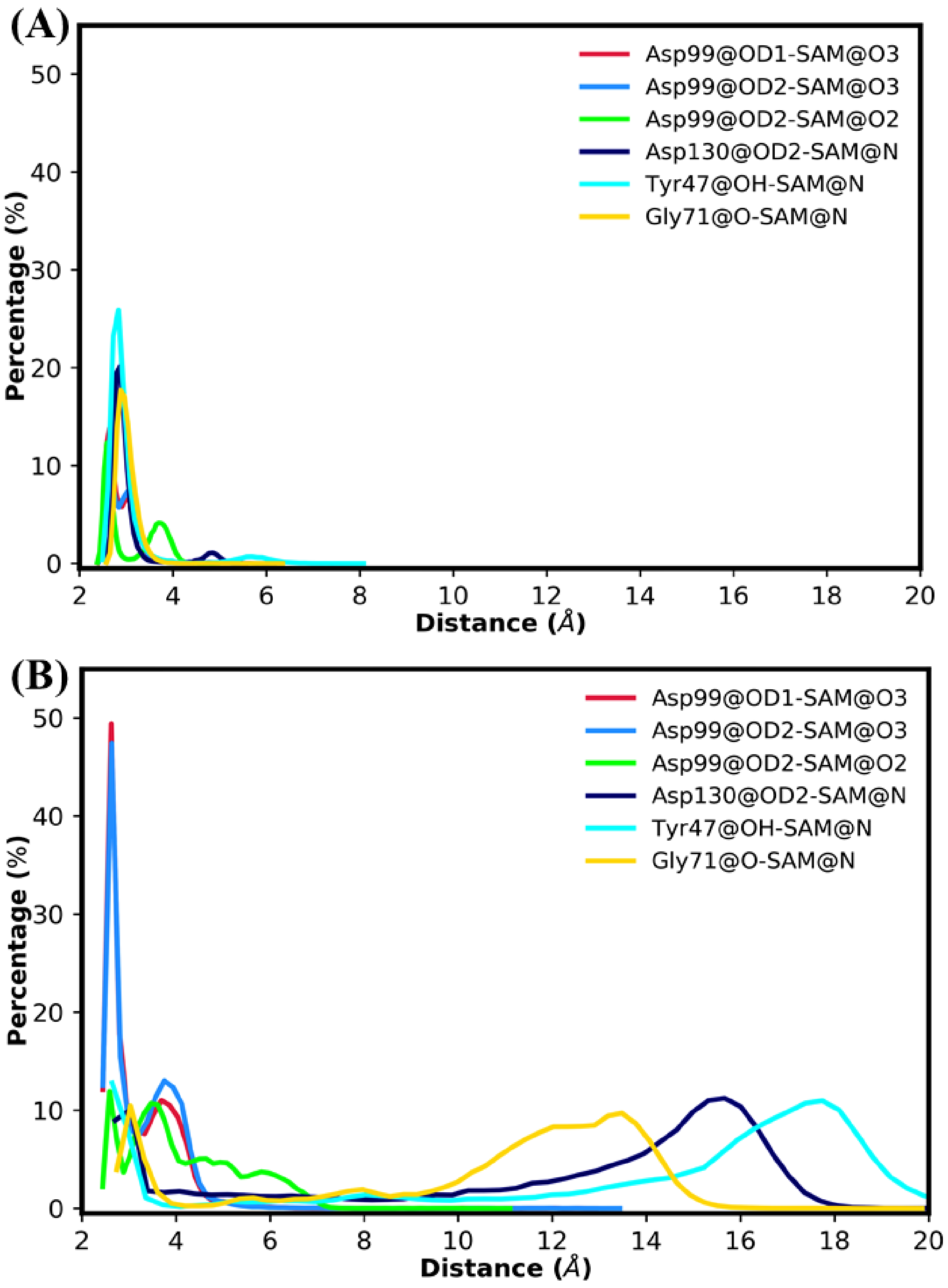

Figure S6: The probability distributions of the hydrogen bonds, (A) complex, and (B) monomer nsp16$_{SAM}$.

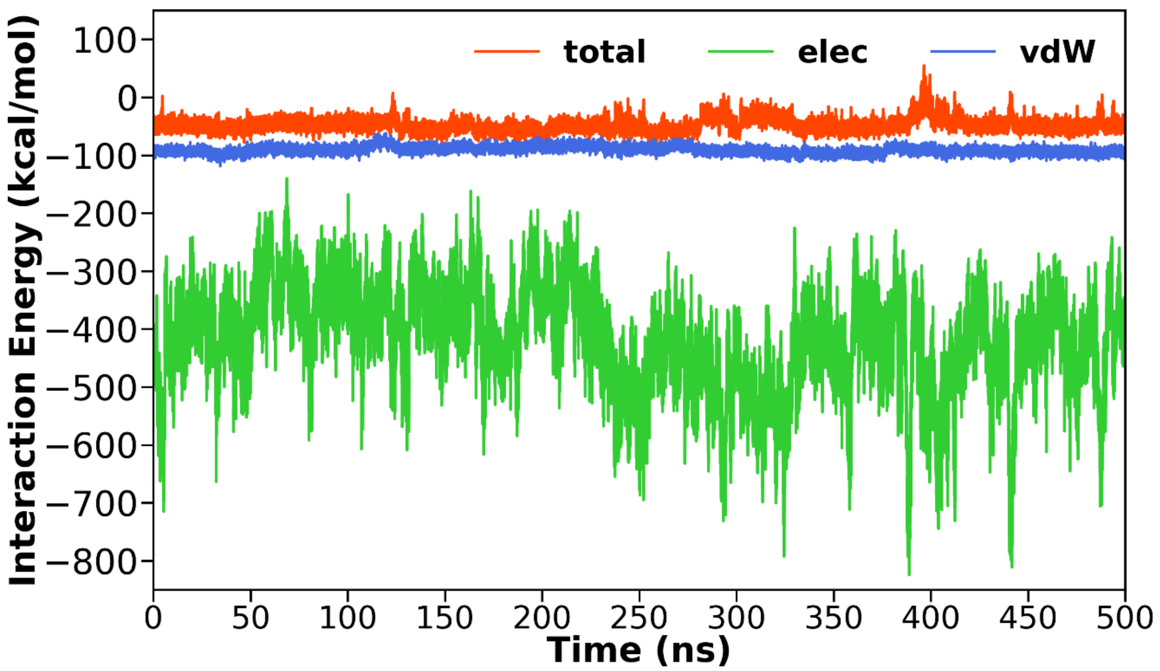

Figure S7: The time evolution of total binding free energy, electrostatic energy, and van der Waals energy in the complex nsp16/nsp10 interaction.

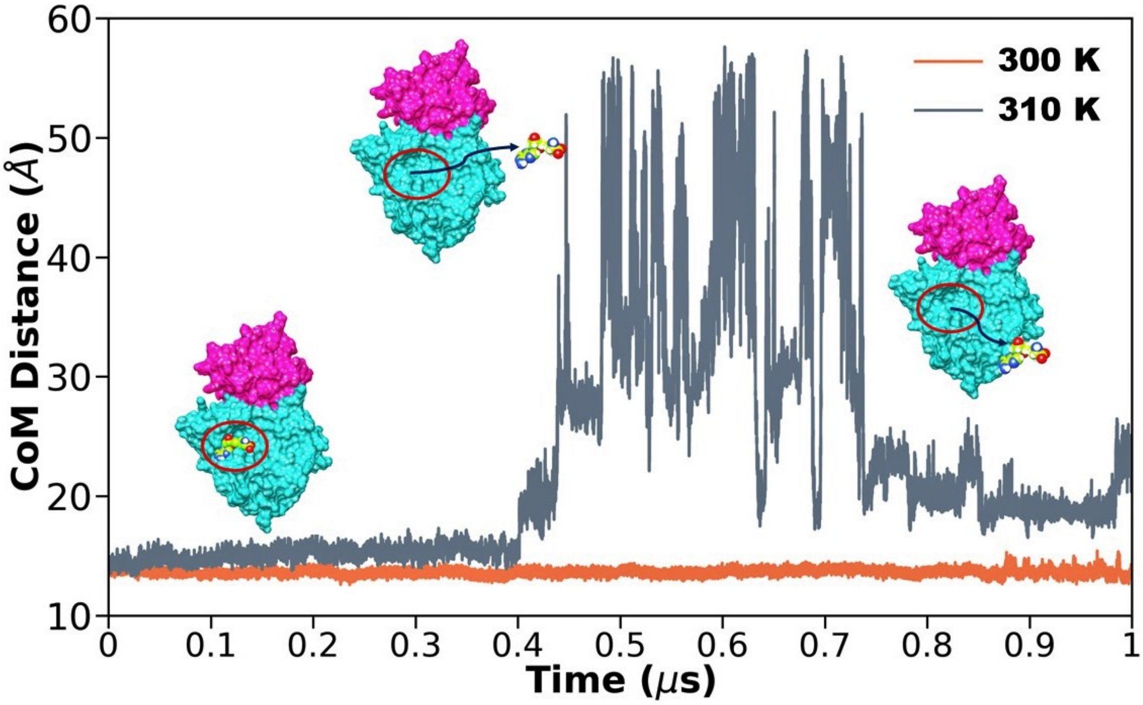

Figure S8: The time evolution of the center of mass (CoM) distance between nsp16 and cofactor SAM at two different temperatures 300 K and 310 K.

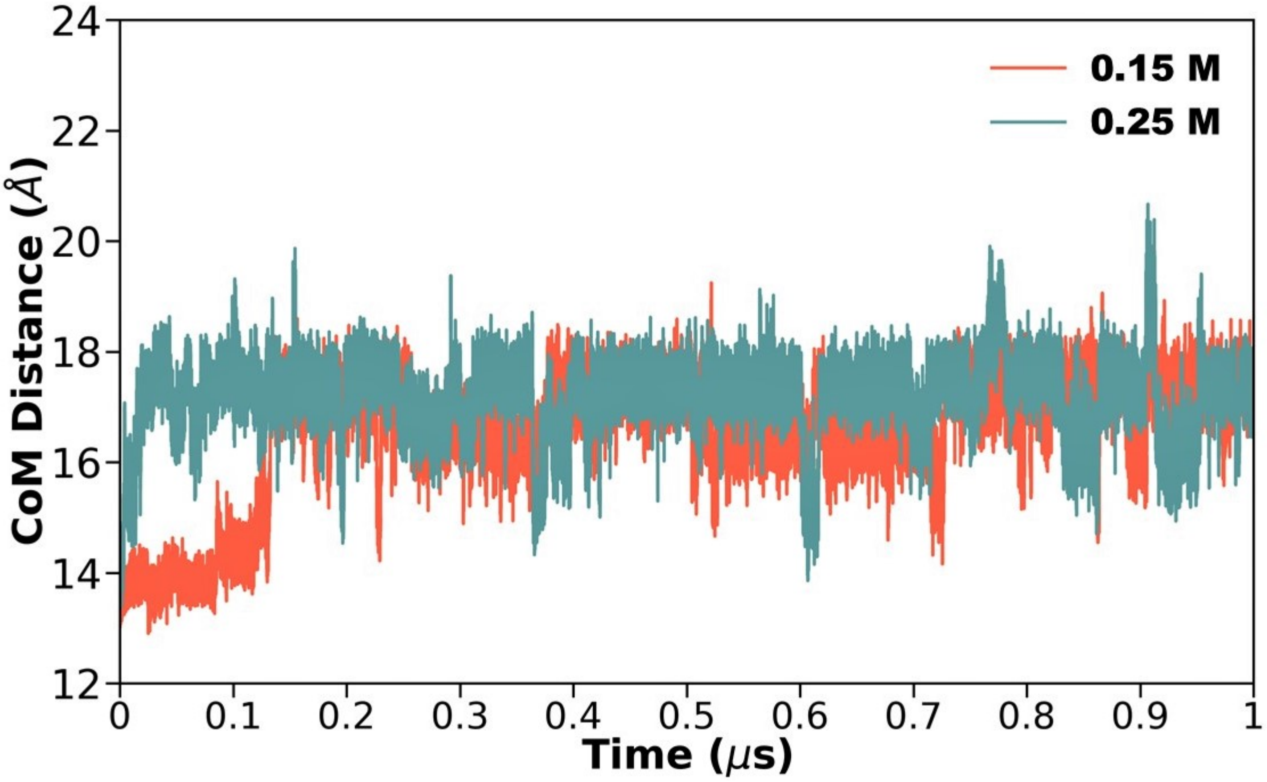

Figure S9: The time evolution of the center of mass (CoM) distance between nsp16 and cofactor SAM at two different salt concentrations, 0.15 M and 0.25 M, respectively.
